# BCG-trained macrophages promote pan-anti-tumor activity through epigenetic rewiring of NOX2-ROS axis

**DOI:** 10.1186/s13046-026-03708-4

**Published:** 2026-04-24

**Authors:** Rui-ming Sun, Yi Yang, Yang-dian Lai, Hai-ning Wang, Xiao-xu Yang, Ping Ji, Ying-ying Chen, Zhao-yuan Liu, Florent Ginhoux, Xiao-yong Fan, Huang-qi Duan, Hai-bo Shen, Dan Ye, Shun Lu, Ying Wang, Zi-ming Li

**Affiliations:** 1 Department of Medical Oncology, Shanghai Chest Hospital, Shanghai Jiao Tong University School of Medicine, Shanghai, 200030 China; 2 Shanghai Institute of Immunology, Department of Immunology and Microbiology, Shanghai Jiao Tong University School of Medicine, Shanghai, 200025 China; 3 Molecular and Cell Biology Laboratory, Institutes of Biomedical Sciences, Shanghai Medical College of Fudan University, Shanghai, China; 4 Department of Urology, Xinhua Hospital, Shanghai Jiao Tong University School of Medicine, Shanghai, 200092 China; 5 Shanghai Institute of Infectious Diseases and Biosecurity & Shanghai Public Health Clinical Center, Fudan University, Shanghai, 201508 China; 6 Shanghai Institute of Virology, Shanghai Jiao Tong University School of Medicine, Shanghai, 200025 China; 7 Translational Immunology Institute, SingHealth Duke-NUS Academic Medical Centre, Singapore, Singapore

**Keywords:** BCG-trained immunity, Tumor infiltrating macrophages, Reactive oxygen species (ROS), NADPH oxidase, Epigenetic reprogramming

## Abstract

**Background:**

Bacillus Calmette-Guerin (BCG)-induced trained immunity in the macrophages is characterized by exaggerated inflammatory cytokine production with favourable effects on disease controls. However, how BCG-trained macrophages exert anti-tumor effects and underlying mechanisms remain to be clarified.

**Methods:**

Pan-anti-tumor activity induced by BCG training was evaluated in mouse models with grafted tumors. The proportion and function of tumor infiltrating macrophages (TAMs) and CD8^+^T cells, as well as the level of reactive oxygen species (ROS), were detected by flow cytometry. The secretion of IL-1β and TNF-α were detected by Enzyme-Linked Immunosorbent Assay (ELISA). The expression and activation of NOX2 complex and NF-κB were detected by qRT-PCR, immunoblotting and immunofluorescence. The methylation modification level and chromatin opening level were detected by CUT&RUN and ATAC sequencing. RNA sequencing was used to detect the transcriptome of macrophages and bladder cancer tissues.

**Results:**

BCG-trained mice exhibited pan-anti-tumor activity where TAMs originated from newly bone marrow hematopoiesis were the predominant effectors. The anti-tumor effects of BCG-trained TAMs were mediated by ROS production in the tumor microenvironment (TME), resulting from the overactivation of NADPH oxidase 2 (NOX2) complex. Epigenetic rewiring of NOX2 complex occurred both in the myeloid progenitor of BCG-trained mice as well as in BCG-trained macrophages marked by increased deposition of H3K4me3 at the promoter regions of NOX2 complex genes, which in turn facilitated the accessibility of transcription factor such as NF-kB and enhanced transcriptional activation. Clinically, NOX2 gene signatures correlated with a favourable prognosis in bladder cancer patients receiving BCG intravesical instillation.

**Conclusions:**

Our findings reveal that BCG training reprograms TAMs to overproduce ROS through epigenetic rewiring of NOX2-ROS axis. Systemic BCG training becomes an effective and promising strategy to remodel the TME with enhanced pan-anti-tumor activity of infiltrating macrophages.

**Supplementary Information:**

The online version contains supplementary material available at 10.1186/s13046-026-03708-4.

## Introduction

Trained immunity confers innate immune cells such as macrophages and nature killer (NK) cells with exaggerated immuno-reactivity upon re-stimulation by unrelated pathogens, thereby mimicking immune memory-like features analogous to those of adaptive immunity [1]. Bacillus Calmette-Guerin (BCG), the only licensed vaccine against *Mycobacterium tuberculosis* (*Mtb*) infection, is one of the classic inducers of trained immunity [2]. BCG was first approved for anti-tumor treatment of non-muscle invasive bladder cancer (NMIBC) through intravesical BCG instillation as the first-line treatment approach [3]. For decades, BCG has also been catalogized as a non-specific biological modulator to treat certain cancers like lung cancer and melanoma [4]. However, the underlying mechanisms remain incompletely addressed.

While CD8^+^ T cells and NK cells act as the main components to exert the cytotoxicity to tumor cells, their anti-tumor efficacy is profoundly influenced by other cells including the macrophages and neutrophils [5]. Notably, macrophages within the tumor microenvironment (TME) exhibit dual and opposing roles in regulating pro- and anti-tumor immunity [6]. To support tumor growth, tumor-associated macrophages (TAMs) can secrete suppressive cytokines such as transforming growth factor-β (TGF-β), interleukin-10 (IL-10), as well as vascular endothelial growth factor (VEGF) to facilitate the angiogenesis [7–9]. They also remodel the extracellular matrix to hinder immune cell infiltration and drive metastasis [10]. Conversely, TAMs also mediate anti-tumor immunity through their inflammatory features [11]. Recent single-cell RNA sequencing (scRNA-seq) analyses have further validated the substantial heterogeneity of TAMs within the TME [12–15]. Given these attributes, TAMs have emerged as a promising target for macrophage-based cancer immunotherapy [16]. Therefore, developing strategies to skew TAM differentiation toward anti-tumor phenotypes will not only impede tumor initiation and progression, but also augment the efficacy of relevant cancer immunotherapy.

Metabolic reprogramming and epigenetic remodeling are key mechanisms that underpin trained immunity in macrophages and NK cells [17]. Specifically, BCG trained macrophages exhibit enhanced activity of glycolysis, pentose phosphate pathway, and TCA cycle [18]. Intermediate metabolites produced by activated glucose metabolism further drive epigenetic modifications such as trimethylation of histone H3 at lysine 4 (H3K4me3) and acetylation of histone H3 at lysine 27 (H3K27ac) at the promoter region of target genes. These modifications ultimately augment pro-inflammatory responses upon secondary stimulation [19]. Notably, hyperactivation of glucose metabolism also promotes the generation of intracellular reactive oxygen species (ROS) [20]. Intracellular ROS supports pro-inflammatory polarization in macrophages, thereby enhancing tumor activity [21–23]. In contrast, excessive ROS also skews the macrophages toward a pro-tumor state [23–25]. Intriguingly, recent studies show that β-glucan, another well-characterized inducer of trained immunity, promotes increased intracellular ROS production in macrophages and neutrophils to exert anti-tumor effects [26, 27]. Given this discrepancy in ROS function and its role in anti- or pro-tumor immunity, whether BCG induced trained immunity mediates anti-tumor effects via ROS generation needs further clarification.

In this study, we demonstrate the pivotal roles of the macrophages in mediating BCG-induced pan-anti-tumor activity. Specifically, BCG-trained TAMs exert anti-tumor effects through elevated ROS production, which is derived from the hyperactivation of the NADPH oxidase 2 (NOX2) complex. Increased deposition of H3K4me3 at the promoter regions of NOX2 complex genes are detectable in BCG-trained macrophages. This epigenetic modification likely facilitates transcriptional accessibility of nuclear factor κB (NF-κB) and thereby promotes excessive ROS generation. In addition, upregulation of NOX2 complex genes correlates with favorable prognosis in bladder cancer patients who receive BCG intravesical instillation. Collectively, our finding elucidates that BCG training reshapes the TAMs within the TME with excessive ROS production and pan anti-tumor activity through epigenetic rewiring of the NOX2-ROS axis, providing a mechanistic foundation to optimize cancer immunotherapies relying on BCG-induced trained immunity.

## Materials and methods

### Cell lines

The LLC, MC38, B16-F10 and RAW264.7 cells were obtained from the Cell Bank of the Chinese Academy of Sciences. SJT-1601 were gifted from *Dr.* Liufu Deng, Shanghai Jiaotong University. MB49 were gifted from *Prof.* Longcheng Li, Peking Union Medical College Hospital. LLC, MC38, B16-F10, MB49 and SJT-1601 cells were maintained in the DMEM medium supplemented with 10% Fetal Bovine Serum (FBS) and 1% Penicillin-Streptomycin (PS). RAW264.7 cells were maintained in the DMEM medium supplemented with 5% Fetal Bovine Serum (FBS), 1% Penicillin-Streptomycin (PS), 1% HEPES buffer, and 1% L-glutamine. During the passage, cells were collected using a pipette into a 15 mL centrifuge tube and centrifuged at 800 rpm for 5 min. Following centrifugation, the cells were gently washed with DMEM prior to either being cultured or proceeding to subsequent analytical steps.

### Mice

Wild-type (WT) (C57BL/6JGpt; N000013) and Nude mice aged 6–8 weeks, were purchased from Shanghai Laboratory Animal Center (Shanghai, China). Ms4a3^***Cre***^_Rosa^***tdTomato***^ mice were gifted from *Dr.* Zhao-yuan Liu, Shanghai Jiao Tong University School of Medicine. The mice were housed in a specific pathogen-free (SPF) facility of Shanghai Jiao Tong University School of Medicine with free access to food and water, and maintained on a 12-hr light/dark cycles at 20–25 °C. Unless stated otherwise, 6–8 weeks old male mice were used in all experiments. The Institutional Animal Care and Use Committee (IACUC) of Shanghai Jiao Tong University School of Medicine (JUMC2023-018-A) approved all animal experiments.

### Human subjects

In total 65 NMIBC patients were recruited in this study from the Department of Urology at Xinhua Hospital affiliated to Shanghai Jiao Tong University School of Medicine (Supplementary Table 1) . All of the participants were initially diagnosed with intermediate-risk or high-risk NMIBC based on the results of the biopsies from cystoscopy examination or TURBt surgery. Among them, all 13 patients received intravesical BCG instillation therapy (120 mg/dose) (Rongsheng, Chengdu, China). BCG instillation therapy included six consecutive instillations each week (induction phase) followed by three weekly instillations at months 3, 6, 12, 18, 24, 30, and 36 (maintenance phase). Cystoscopy and biopsies were conducted under regional anesthesia 6 weeks after each treatment phase. Based on the results from cystoscopy and biopsies, 9 patients who showed no recurrence at 6 months from the beginning of the treatment were defined as “BCG responder”, and 4 underwent the recurrence at 6 months that were defined as “BCG non-responder”. And 2 of the “BCG non-responder” received cystoscopy surgery again at 6 months that were defined as “BCG recurrence”. All patients signed informed consent forms. This study was approved by the Ethical Committee of Xinhua Hospital, and the procedures performed in this study were in accordance with the 1964 Helsinki declaration.

### Bacterial culture

BCG used in this study is Japan strain and were gifted from *Prof.* Xiaoyong Fan (Fudan University, Shanghai, China) and grown in 7H9 broth (BD) supplemented with 0.2% glycerol (Wisent), 0.05% Tween80 (Fisher), and 10% albumin-dextrose-catalase (ADC) under constant shaking at 37 °C.

### Preparation of bone marrow derived macrophages

Bone marrow was extracted from the femurs and tibias of the mice using PBS enriched with 2% FBS. A total of 30 million bone marrow cells were seeded into 10 mL cell culture medium that consisted of the following components: 10% FBS, 1% L-glutamine, 1% HEPES buffer, 1% MEM Non-Essential Amino Acids Solution, 1% PS, 0.1% 2-mercaptoethanol, and 50 ng/mL of Macrophage Colony-Stimulating Factor (M-CSF). The cells were incubated for 6 days in a 37 °C humid condition with 5% CO_2_. At day 3, half of the culture medium was replaced with DMEM culture medium. At day 6, bone marrow-derived macrophages (BMDMs) were labeled with CD11b (APC) and F4/80 (bv421) and using flow cytometry to show a purity of approximately 90% for further studies.

### BCG treatment in vitro and in vivo

For in vitro experiments, BMDMs and RAW264.7 cells were treated with BCG lysate (10 µg/mL) for 24 h and washed once with DMEM culture medium. Cells were rested for 3–5 days and subjected to further experiments. BCG lysate was prepared by the sonication of fresh BCG bacillus. For in vivo experiments, BCG bacillus (OD = 0.8 ~ 1.0) were centrifuged at 500 g for 2 min. Bacillus pellets were resuspended in PBS. Mice were intraperitoneal (*i.p.*) injected with either PBS or BCG (6 × 10^6^ CFU/200 µL).

### Mouse tumor models

For tumor models, tumor cells were injected subcutaneously (s.c.) into 8-week-old mice (5 × 10^5^ LLC cells, 5 × 10^5^ MC38 cells, 2 × 10^5^ B16-F10, 1 × 10^5^ MB49 cells and 5 × 10^5^ SJT-1601 cells per mouse). Tumor volumes were monitored from Day 7 and were calculated by using the equation (length x width^2^) / 2. Calculation of tumor volumes was done in a blinded fashion and the lethality was defined as tumor volumes reaching 1500 mm^2^.

### In vivo cell depletion

For In vivo depletion of CD8^+^T cells, 250 µg/100µL purified anti-CD8 monoclonal antibodies (2.43, BioXCell) were intraperitoneal (*i.p.*) injected on day − 1, 6 and 13 respectively and 5 × 10^5^ LLC cells were injected on Day 0.

For In vivo depletion of macrophages or NK cells, 250 µg/100µL purified anti-F4/80 monoclonal antibodies (CI: A3-1, BioXCell) or anti-NK1.1 monoclonal antibodies(PK136, BioXCell) were intraperitoneal (*i.p.*) injected on day − 1, 4, 9 and 14 respectively and 5 × 10^5^ LLC cells were injected on Day 0. For In vivo depletion of neutrophil, 150 µg/100µL purified anti-Ly6G monoclonal antibodies (1A8, BioXCell) were intraperitoneal (*i.p.*) injected on day − 1, 2, 5, 8, 10 and 15 respectively and 5 × 10^5^ LLC cells were injected on Day 0. Control mice were treated with *i.p.* injection of 100 µL PBS.

### Preparation of mouse tumor infiltrating lymphocytes

Grafted tumors were minced into small pieces and put into 1.5 mL centrifugal tubes followed by enzymatic digestion in FBS-free DMEM culture medium containing 16 mg/mL Enzyme D, 0.4 mg/mL Enzyme R and 0.5 mg/mL Enzyme A (all from MiltenyiBiotec). Cell suspensions were successively filtered through 70 mm cell strainers (MiltenyiBiotec) to obtain single cell suspension and subject to further experiments.

### Flow cytometry

For CD8^+^T cells and NK cells detection, mouse TILs were treated with PMA(0.1 µg/mL, Sigma), Ionomycin (1 µg/mL, ENZO) and GolgiStopTM (0.1%, BD Biosciences) for 4 h.Then cells were stained in PBS containing 2% FBS (FACS buffer) with FVS (BV510) and antibodies targeting CD45 (AF700), CD3(bv711), CD8(PE-cy7) and NK1.1(Bv650) incubated for 30 min at 4 °C, washed once with FACS buffer. Then cells were fixed and permeabilized by using Cytofix/Cytoperm™ Kit (BD Biosciences) according to the manufacturer’s instructions. Flowing intracellular straining targeting IFN-γ(Apc-cy7),TNF-α (APC), CD107A(FITC) and Granzyme B(PE).

For macrophages and neutrophils detection, mouse TILs were stained in PBS containing 2% FBS (FACS buffer) with FVS (BV510) and antibodies targeting CD45 (AF700), CD11b(APC), F4/80(Bv421), Ly6G(Bv786), Ly6C(Bv605), CD80(FITC), CD86(PE) and MHC-II(Percp-cy5.5) incubated for 30 min at 4 °C, washed once with FACS buffer. Then cells were fixed and permeabilized by using Cytofix/Cytoperm™ Kit (BD Biosciences) according to the manufacturer’s instructions. Flowing intracellular straining targeting CD163(Apc-cy7) and CD206(Bv650).

For TAM1/2/3, Ms4a3 and cytokines of macrophages detection, mouse TILs were stained in PBS containing 2% FBS (FACS buffer) with FVS (Bv510) and antibodies targeting CD45 (Apc-cy7), CD11b(Buv395), F4/80(Bv421), Ly6G(Bv786), Ly6C(Bv605), MHC-II (AF700), CD64 (Bv711), CCR2 (APC), CD73(Percp-cy5.5) and CD11c(FITC) incubated for 30 min at 4 °C, washed once with FACS buffer. Then cells were fixed and permeabilized by using Cytofix/Cytoperm™ Kit (BD Biosciences) according to the manufacturer’s instructions. Flowing intracellular straining targeting pro-IL1β (PE-cy7) and TNF-α(Bv650). The Ms4a3-tdTomato was detected by PE channel.

For hematopoietic cells, mouse bone marrow single-cell suspension were stained in PBS containing 2% FBS with lineage(Streptavidin) and antibodies targeting Streptavidin(Percp-cy5.5), Sca-1(FITC), C-Kit(APC), CD48(Bv510), CD150(PE-cy7), CD34(ef450) and CD16/32(PE) for 30 min at 4 °C, washed once with FACS buffer.

For BMDMs and Raw264.7 cells ROS detection, cells were firstly incubated with highly sensitive DCFH-DA (1:1000, Dojindo) or mitoSOX(10µM, Thermo) for 30 min at 37 °C, then washed and labeled with CD11b (APC) and F4/80 (bv421) for 30 min at 4 °C.

Cells were acquired on BD LSR Fortessa X20 (Beckton Dicknson) for analyzing and on BD FACS Arialll for sorting. Data were analyzed with FlowJo Version 10.0 software.

### Transwell assay

PBS or BCG trained BMDM were inoculated into the upper chamber of the transwell system with the number of 50,000/well, while LLC cells were inoculated into the lower chamber the number of 25,000/well. DMSO or CCR2 inhibitor PF-4,136,309 were added and the cells were incubated for 48 h. Afterwards, flow cytometry was used to detect the number of BMDMs(F4/80^+^ CD11b^+^) in the lower chamber.

### Immunofluorescence assay (IFA)

BMDMs were cultured on 12-well plates with cover slips. They were then fixed with 4% PFA for 15 min at room temperature. Afterward, cells were permeabilized with 0.1% TritonX-100 for 10 min. After blocking with 3% BSA at room temperature for 20 min, the cells were incubated with primary antibodies targeting NF-κB p65 at 4 °C overnight and incubated with fluorescence-labeled secondary antibodies at 37 °C for 1 h. The cells were incubated with DAPI in the dark for 5 min.

The paraffin section of the patient’s bladder cancer was dewaxed in 95 − 75% ethanol, and then antigen repair was performed. After blocking with 3% BSA at room temperature for 30 min, they were incubated overnight at 4 °C for the corresponding primary antibody: CD68(abcam), p22^phox^(affinity) and NOX2(abcam).The fluorescence-labeled secondary antibodies were incubated at 37 °C for 1 h and then incubated with DAPI in the dark for 5 min.

Confocal images were captured using a DeltaVision OMX SR system, and the images were aquired by using ImarisViewer 10.0.1 software. Fluorescence quantitative statistical analysis was performed using Image J software.

### Western blotting

The cells were washed with ice-cold PBS and subsequently lysed on ice for 30 min in RIPA lysis and extraction buffer supplemented with phosphatase inhibitor cocktail (1 mM, TargetMol) and protease inhibitor cocktail (1 mM, TargetMol). Cell lysates were collected through centrifugation at 15,000 rpm for 10 min, and protein concentrations were determined by using the BCA assay (Beyotime Biotechnology). Cell lysates were then mixed with Omni-Easy™ instant protein loading buffer (5X) and subjected to 10% sodium dodecyl sulphate-polyacrylamide gel electrophoresis and subsequently transferred to polyvinylidene difluoride (PVDF) membranes (Millipore) using a transfer system (Bio-Rad). The membranes were blocked with 1X protein-free Rapid Blocking Solution (Epizyme) for 30 min at room temperature, followed by overnight incubation at 4 °C with primary antibodies p-p67^phox^(Affinity Biosciences,1:1000), NOX2(Affinity Biosciences,1:1000), p22^phox^(Affinity Biosciences,1:1000), RAC1(CST,1:1000), p-NF-κB p65(Beyotime Biotechnology,1:500) and β-actin(CST,1:5000). Following a 1 h incubation with horseradish peroxidase-conjugated secondary antibodies, target proteins were visualized using the ECL detection system (Tanon). p67^phox^ (AiFang biological,1:1000) or NF-κB p65(CST, 1:1000) were incubated after the completion of p-p67^phox^ or p-NF-κB p65 development and incubation with rapid release buffer for 15 min in the same membranes.

### RNA interference and reverse transcription-PCR (qRT-PCR)

Raw264.7 cells were transfected with *mCyba*-specific siRNA (50 nM) or *mCybb*-specific siRNA (50 nM) by using CALNP™ mRNA In Vitro commercial kit (D-Nano Therapeutics) according to the manufacturer’s protocol while a ‘nonsense’ sequence served as the negative control. To determine the expression of target genes, total RNA was extracted after 48 h by using the TRIzol reagent (Invitrogen) following the protocol provided by the manufacturer and reversely transcribed into cDNA by PrimeScript Reverse Transcriptase (Takara). Polymerase chain reaction (PCR) was performed by using TB Green^®^ Premix Ex Taq™ II (Tli RNase H Plus) (Takara) on an Applied Biosystems ViiA™ 7 Real-Time PCR system. Gene expression levels in individual samples were calculated based on the 2^−△△^CT according to the threshold cycle (CT) values of target genes and house-keeping gene. Primers were listed in Primers and probes in Supplementary Table 2.

### ELISA

TNF-α and IL-1β contents in culture supernatants were determined by using the Mouse TNF-α ELISA Kit (Thermo) and Mouse IL-1β ELISA Kit (Thermo) following the manufacturer’s instructions.

### CUT&RUN assay

BMDMs were trained with PBS or BCG for 24 h, then the supernatant were washed and added new complete culture medium or medium containing 10µM MM102 for 2–3 days. Cells were collected and subjected to CUT&RUN assay to evaluate the enrichment level of H3K4me3 modification in the promoter regions of *Cyba*, *Cybb*, and *Ncf2* genes. CUT&RUN assay were performed using Vazyme’s reagent kit, strictly following the manufacturer’s instructions. IgG were selected as a negative control. The primers involved in CUT&RUN assay were listed in Primers and probes in Supplementary Table 2.

### Bulk RNA-sequencing and data analysis

Total RNA was extracted using the TRIzol reagent (Invitrogen) following the manufacturer’s protocol. RNA purity and quantification were assessed using the NanoDrop 2000 spectrophotometer (Thermo Scientific). RNA integrity was evaluated using the Agilent 2100 Bioanalyzer (Agilent Technologies). The libraries were constructed using the VAHTS Universal V6 RNA-seq Library Prep Kit following the manufacturer’s instructions. The transcriptome sequencing analysis were performed by Majorbio Co., Ltd. (Shanghai, China). The libraries were sequenced on an Illumina Novaseq 6000 platform, generating 150 bp paired-end reads. FPKM3 values for each gene were calculated, and the read counts were obtained using HTSeq-count4. Differential expression analysis was conducted using DESeq2 5. A P value < 0.05 and foldchange > 1.5 or foldchange < 0.5 were set as thresholds for significantly differentially expressed genes (DEGs).

Pathway enrichment was calculated using several biological databases (KEGG, Reactome, Biocarta, and Panther) with an FDR p-value < 0.05. For gene set enrichment analysis (GSEA) analysis, GOBP_REACTIVE_OXYGEN_SPECIES_BIOSYNTHETIC_PROCESS and HALLMARK_TNFA_SIGNALING_VIA_NFKB were performed using GSEA based R package.

### ATAC-sequencing and data analysis

ATAC-seq experiment was performed as instructions with minor modifications. Briefly, mouse hematopoietic progenitor cells from bone marrows were purified by Mouse Hematopoietic Progenitor Cell Enrichment following the instructions. After that, cells were stained in FACS with antibodies targeting lineage(Streptavidin), Streptavidin(Percp-cy5.5), Sca-1(FITC), C-Kit(APC), CD48(Bv510), CD150(PE-cy7) and short-term hematopoietic stem cells (ST-HSCs) were sorted by BD FACS Arialll. About 10,000 ST-HSC or BMDM cells per experiment were washed for twice and resuspended in lysis buffer [1 M Tris-HCl (pH 7.4), 5 M NaCl, 1 M MgCl_2_, 10% NP-40, 10% Tween-20, and 1% Digitonin] for 5 min on ice. Then, cells were suspended in 50 µL of Tn5 tagmentation master mix and incubated at 37 °C for 30 min. Tagmented DNA fragments were purified with DNA Extract Beads (Vazyme). The library was amplified for 15 cycles, purified with DNA Clean Beads (Vazyme) by size selection, and sequenced on illumina Next-seq platform running in PEx150bp mode.

The reads were trimmed with Trim-galore (version 0.6.10) and aligned to the mouse reference genome (mm10) with Bowtie2 (version 2.5.1) using the option -X 2000. Then, reads with improperly paired, mapped to chrY and mitochondria, and alignment quality < Q20 were discarded. Duplicated reads were removed using *MarkDuplicates* function of Picard (https://broadinstitute.github.io/picard/). MACS2 (version 2.2.7.1) was used to call narrow peaks with the following parameters (-B --SPMR --nomodel --shift − 75 --extsize 150 --call-summits). Peaks overlapping with blacklisted regions (https://github.com/Boyle-Lab/Blacklist/tree/master/lists) were filtered out using bedtools (version 2.30.0). Count per million (CPM) was calculated for normalization and differential peak identification. Peaks that were enriched (fold change > 2, FDR < 0.05) were considered to gain chromatin accessibility. Heatmaps were generated with deeptools (version 3.5.1). Peaks annotation and motif analysis were performed using *annotatePeaks* and *findMotifsGenome* script of Homer (version 4.11). The track plot was visualized by Integrative Genomics Viewer (IGV) (version 2.18.2).

### Single cell RNA-sequencing data collection

This study integrates human- and mouse-derived single-cell RNA sequencing (scRNA-seq) data for comparative analysis. The human data were obtained from urine-derived cells (UDCs) collected from a bladder cancer patient at two time points: one week before intravesical BCG instillation (w1pre) and six weeks post-instillation (w6post). The corresponding scRNA-seq data were acquired from the Gene Expression Omnibus (GEO accession number GSE267718, with sample IDs GSM8273677 and GSM8273685, respectively). For the murine data, we employed the MB49 bladder tumor model in C57BL/6 mice, where the experimental group received BCG treatment while the control group was administered PBS. Tumor-infiltrating CD45^+^ immune cells were subsequently isolated for scRNA-seq analysis, with the sequencing data deposited in the GEO dataset GSE295309 (sample IDs GSM8939888 and GSM8939889). This parallel human-mouse experimental design provides cross-species evidence for elucidating the immunomodulatory mechanisms of BCG therapy in the bladder cancer microenvironment.

### scRNA-seq data processing

During the data processing phase, the raw data from the two samples were merged, and cells expressing more than 20% mitochondrial genes were excluded to ensure data quality. Based on the distribution characteristics of each sample, cutoff values for gene counts and unique molecular identifiers (UMI) counts were determined. Batch effects were corrected using the BBKNN algorithm to ensure data consistency. Subsequently, the data underwent normalization and log transformation with addition of 1, and high-variance genes (HVGs) were identified using the Scanpy v1.9.1 software package. Thereafter, cluster analysis was performed using the Leiden algorithm with a resolution set at 0.5, and uniform manifold approximation and projection (UMAP) technology was utilized for data dimensionality reduction and embedding. For each broad cell type, HVGs were recalculated, and cells were re-clustered at a higher resolution to achieve a more refined separation of cell types.

### Cell subtype and gene expression analysis

Using UMAP dimensionality reduction analysis, we performed comprehensive cellular classification and molecular characterization across two distinct biological systems. In human urine-derived cells (UDCs), we identified seven phenotypically distinct subpopulations: (1) KRT18+/KRT19 + urothelial cell subtype 1, (2) KRT16+/KRT19 + urothelial cell subtype 2, (3) CD3 + T lymphocytes, (4) CD14 + macrophages, (5) FCGR3B+ neutrophils, (6) MS4A2+/LTC4S+ mast cells, and (7) KLRK1+/FCGR3A+ natural killer cells. Parallel analysis of murine tumor microenvironment revealed seven characteristic immune cell subsets: (1) Cd79a + B lymphocytes, (2) Flt3+/Zbtb46 + dendritic cells, (3) Ly6c2 high monocytes, (4) Mrc1+/Cd68 + tumor-associated macrophages, (5) Krt18 + tumor epithelial cells, (6) Cd3e + T lymphocytes, and (7) S100a8 high neutrophils.

In the process of gene expression analysis for macrophage subtypes, our research team initially isolated macrophages using the specific surface marker ‘CD14’ or ‘Cd68’. Subsequently, we employed the ‘rank_genes_groups’ function from the scanpy package, utilizing the ‘wilcoxon’ method to analyze the gene expression data of two groups of macrophages. We determined the differentially expressed genes (DEGs) by calculating the Log2 Fold Change (LFC) and p-value of the average gene expression in macrophages from the two samples. The Log2 Fold Change is a measure of the logarithmic fold change in gene expression between two conditions, while the p-value assesses the statistical significance of this change. Compared to w1pre, genes with an LFC greater than 1 and a p-value less than 0.05 were classified as upregulated DEGs, whereas genes with an LFC less than − 1 and a p-value less than 0.05 were considered downregulated DEGs.

### Quantification and statistical analysis

The data were presented as mean ± standard error of the mean (SEM). Statistical analysis was performed using Prism 10.0 software. Statistical significance was determined by unpaired *Student’s* t test, One-way ANOVA, two-way ANOVA and is represented by the following scheme: *p < 0.05, **p < 0.01, ***p < 0.001 and ****p < 0.0001.

## Results

### BCG-trained mice display pan-anti-tumor activity relying on the macrophages

BCG is one of the representative agents to induce trained immunity in the macrophages, which is manifested by elevated production of pro-inflammatory cytokines such as IL-1β and TNF-α upon virus or bacteria re-stimulation [2, 28, 29]. To investigate the impacts of BCG-induced trained immunity on anti-tumor responses, we firstly established a BCG-trained mouse model. Unlike the previous report describing a BCG-trained mouse model through intravenous (*i.v.*) injection of BCG [30], we intraperitoneally (*i.p.*) injected 6 × 10^6^ colony forming units (cfu) of BCG bacilli to C57BL/6 mice. Seven days later, the splenocytes were re-stimulated with LPS in vitro (Supplementary Fig. 1A). It was found that the macrophages from BCG-treated mice produced significantly higher levels of TNF-α and pro-IL1β than those from PBS treated mice (Supplementary Fig. 1B-1C), recapitulating trained immunity features analogous to those observed in the *i.v.* induced model.

**Fig. 1:**
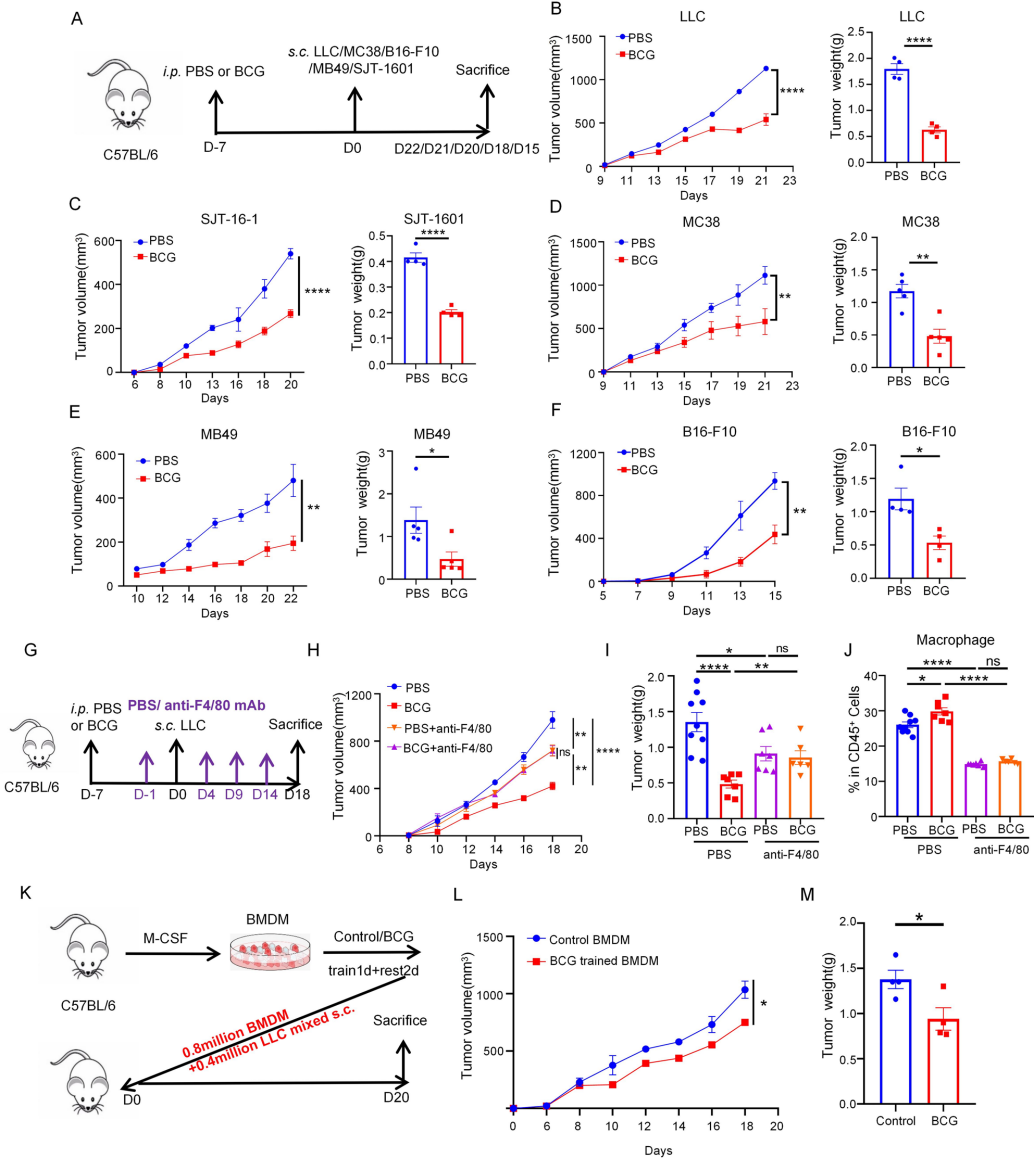
BCG-trained mice display pan-anti-tumor activity relying on the macrophages. **A** Experimental scheme. Briefly, BCG was intraperitoneally injected once and multiple subcutaneous grafted tumors were established at Day 7. Tumor growth curves were monitored and mice were sacrificed according to tumor volumes. **B** Tumor growth curves of grafted Lewis lung carcinoma (LLC) (left) and tumor weight (right) in PBS (*n* = 4) versus BCG treated (*n* =4) mice. **C** Tumor growth curves of grafted mouse lung cancer cell line SJT-1610 (left) and tumor weight (right) in PBS (*n* = 4) versus BCG treated (*n* =4) mice. **D** Tumor growth curves of grafted mouse colorectal cancer cell line MC38 (left) and tumor weight (right) in PBS (*n* = 5) versus BCG treated (*n* =5) mice. **E** Tumor growth curves of grafted mouse bladder cancer cell line MB49 (left) and tumor weight (right) in PBS (*n* = 5) versus BCG treated (*n* =5) mice. **F** Tumor growth curves of grafted mouse melanoma cell line B16-F10 (left) and tumor weight (right) in PBS (*n* = 4) versus BCG treated (*n* =4) mice. **G** Working schemes of the macrophage depletion experiments. Briefly, BCG was intraperitoneally injected once. Anti-mouse F4/80 antibody (Ab) was injected one day before (Day -1), Day4, Day9 and Day 14 after subcutaneous grafted tumor injection. Tumor growth curves were monitored and mice were sacrificed at Day 18. **H**-**J** Comparisons of tumor growth curves (**H**), tumor weight (**I**) and the percentages of macrophages in CD45+ cells in LLC grafted tumors (**J**) among PBS (*n* = 9), BCG-treated (*n* = 7), PBS+anti-F4/80 Ab (*n* = 7) and BCG-treated + anti-F4/80 Ab (*n* = 6) group mice. Data from two independent experiments were pooled and presented. **K** Experimental scheme of co-transferring of PBS or BCG-trained BMDMs and tumor cells in C57BL/6 mice. **L**-**M** Comparisons of tumor growth curves (**L**) and tumor weight (**M**) of LLC grafted tumors in control and BCG-trained BMDM transferred mice (*n* = 4). Data are presented as mean ± SEM. ns: non-significant, * *p* < 0.05, ** *p* < 0.01, **** *p* < 0.0001 by the Student’s t test (**B**-**F**,**M**) and One-way ANOVA (**H**-**J**). Unless indicated, data were the representative of two independent experiments

Given the well-established role of macrophages in regulating anti-tumor immunity, we next sought to determine whether BCG-induced trained immunity confers pan-anti-tumor activity. BCG-trained C57BL/6 mice were subcutaneously inoculated with multiple mouse tumor cell lines. Tumor volumes were measured every two days until the mice were sacrificed (Fig. 1A). We found that compared to PBS treated control mice, BCG treatment significantly retarded the growth of grafted tumors in vivo with reduced tumor weight after the sacrifice, including the lung cancer with mouse Lewis lung carcinoma (LLC) (Fig. 1B) and SJT-1601 (Fig. 1C), the colon cancer with mouse cell line MC38 (Fig. 1D), the bladder cancer with mouse cell line MB49 (Fig. 1E), and the melanoma with mouse cell line B16-F10 (Fig. 1F). To further validate the persistence of anti-tumor immunity elicited by BCG-induced trained immunity, we subcutaneously inoculated LLC tumor cells at 6 weeks after *i.p.* BCG injection (Supplementary Fig. 1D). Our results showed that BCG injection still exerted an inhibitory effect on tumor growth (Supplementary Fig. 1E-1F). Therefore, intraperitoneal pre-injection of BCG induced durable pan-anti-tumor activity in the mice.

To further identify the immune cell subsets responsible for BCG-induced enhanced anti-tumor effects, we performed cell depletion experiments targeting macrophages, CD8^+^T cells, natural killer (NK) cells, neutrophils et al. Anti-F4/80 monoclonal antibodies (mAb) were administered concurrently with the inoculation of tumor cells in BCG-trained or PBS treated mice (Fig. 1G). We found that BCG-treated mice receiving anti-F4/80 mAb accelerated tumor growth when compared to control group mice (Fig. 1H, red and purple) and increased tumor weight after the sacrifice (Fig. 1I, red and purple), indicating that elevated anti-tumor activity induced by BCG partially depends on the macrophages. Consistently, less tumor infiltrating macrophages were detectable after anti-F4/80 mAb treatment (Fig. 1J). Notably, while anti-F4/80 treatment did not diminish the frequency of tumor-infiltrating CD8⁺ T cells in BCG-trained mice (Supplementary Fig. 1G-1H), it significantly attenuated cytokine production such as IFN-γ and TNF-α (Supplementary Fig. 1I–1J). Cytotoxic markers such as CD107A and granzyme B were also down-regulaed (Supplementary Fig. 1K–1L), which implies macrophage-dependent functional priming of CD8⁺ T cells. To assess T cell dependency, BCG or PBS was administered to nude mice followed by MC38 tumor grafting (Supplementary Fig. 2A). Compared to wild-type mice, both BCG- and PBS-treated nude mice showed accelerated tumor growth (Supplementary Fig. 2B–2D). Furthermore, we used anti-CD8 monoclonal antibody to deplete CD8^+^T cells (Supplementary Fig. 2E), which was consistent with the results in nude mice. The depletion of CD8^+^T cells weakened the anti-tumor effect induced by BCG to some extent, but this difference could not be completely eliminated (Supplementary Fig. 2F–2H), ruling out a critical role for T cells in BCG-mediated anti-tumor activity. When we used anti-NK1.1 mAb (Supplementary Fig. 2I) and anti-Ly6G mAb (Supplementary Fig. 2M) to deplete NK cells and neutrophils respectively, no significant difference in tumor growth and tumor weight was observed between mAb-treated and mAb-naïve BCG-trained mice (Supplementary Fig. 2J-2L and 2N-2P) indicating that these cell types are dispensable for BCG-induced exaggerated anti-tumor effects. Interestingly, depletion of NK cells and neutrophils led to the increase in tumor weight in PBS-treated mice.

To further verify the critical roles of the macrophages in enhanced anti-tumor immunity after BCG induction, we generated BCG-trained bone marrow derived macrophages (BMDMs) in vitro. BMDMs were stimulated with BCG lysate or control medium for 24 h. After washing and resting for 3 days, we performed the re-stimulation with LPS (Supplementary Fig. 3A). It was found that BCG-treated BMDMs produced more TNF-α and IL-1β as compared to the BMDMs without BCG treatment (Supplementary Fig. 3B-3C). Next, we mixed in vitro BCG-trained or non-trained BMDMs with LLC tumor cells and inoculated subcutaneously into WT mice (Fig. 1K). It was found that grafted tumor growth was retarded with BCG-trained BMDMs (Fig. 1L) and tumor weight was significantly declined (Fig. 1M). We also co-cultured BCG-trained BMDMs with LLC and MC38 tumor cells in vitro, and measured the contents of lactate dehydrogenase (LDH) in the supernatants to indirectly determine the cytotoxicity efficiency of the macrophages against tumor cells (Supplementary Fig. 3D). Our results showed that BCG-trained BMDMs exhibited stronger cytotoxicity to both LLC and MC38 tumor cells (Supplementary Fig. 3E). In addition, mouse macrophage cell line Raw264.7 after BCG treatment also exhibited the phenotypes of trained immunity with increased production of TNF-α and IL-1β after LPS re-stimulation (Supplementary Fig. 3F-3 H) and enhanced the cytotoxicity to LLC and MC38 cells (Supplementary Fig. 3I-3 J). Collectively, these findings establish macrophages as the key player in enhanced pan-anti-tumor activity in BCG-trained mice.

### BCG-trained macrophages acquire a pro-inflammatory TAM1-like phenotype with more capacity to infiltrate into the TME

**Fig. 2:**
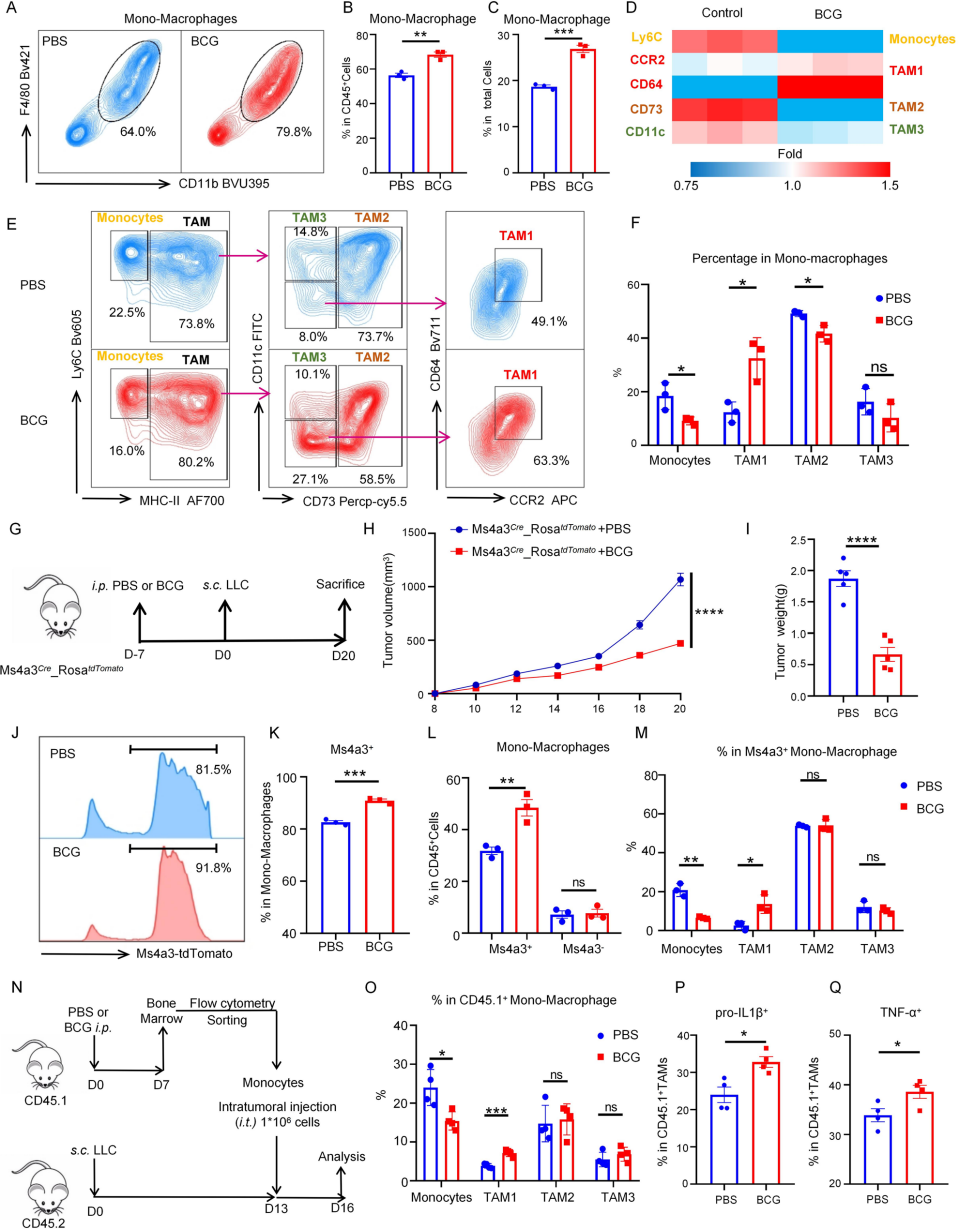
BCG-trained macrophages acquire a pro-inflammatory TAM1-like phenotype with more capacity to infiltrate into the TME. **A**-**C** Representative flow contour maps (**A**), and comparisons of the percentages of mono-macrophages in CD45+ cells (**B**) and in total cells (**C**) in LLC grafted tumors between PBS and BCG-treated mice. **D** Heatmaps of Ly6C, CCR2, CD64, CD73 and CD11c expression levels of control BMDM and BCG-trained BMDMs by flow cytometry (*n*= 3). **E**-**F** Representative flow contour maps (**E**) and comparisons of the percentages of monocytes and TAM subsets (**F**) in LLC grafted tumors between PBS (*n*=3) and BCG-treated mice (*n*=3). **G** Experimental scheme. Briefly, BCG was intraperitoneally injected once and LLC grafted tumors were established at Day 7 in Ms4a3-tdTomato reporter mice. Tumor growth curves were monitored and mice were sacrificed according to tumor volumes. **H**-**I** Comparison of grafted LLC tumor growth curves (**H**) and tumor weight (**I**) between PBS and BCG-treated Ms4a3^*Cre*^_Rosa^*tdTomato*^ mice (*n*= 5 per group). Data from two independent experiments were pooled and presented. **J**-**K** Infiltration of Ms4a3+ mono-macrophages (**J**) and comparisons of Ms4a3+ percentages in mono-macrophages (**K**) in LLC grafted tumors from PBS and BCG-treated mice (*n*= 3 per group). **L** Comparisons of the percentages of Ms4a3+ and Ms4a3- mono-macrophages in CD45+ cells in LLC grafted tumors from PBS and BCG-treated mice (*n*= 3 per group). **M** Comparisons of the percentages of monocytes and TAM subsets in Ms4a3+ mono-macrophages in LLC grafted tumors from PBS and BCG-treated mice (*n*= 3 per group). **N** Experimental scheme. Briefly, PBS or BCG were intraperitoneally injected to CD45.1 mice. After 7 days injection, the sorting monocytes were intratumoral injected into CD45.2 LLC tumor grafted mice at Day 13. After 3 days, mice were sacrificed and tumor were dissect and performed flow cytometry analysis. **O** Comparisons of the percentages of monocytes and TAM subsets in CD45.1+ mono-macrophages in LLC grafted tumors (*n*= 3 per group). **P**-**Q** Comparisons of the percentages of pro-IL1β (**P**) and TNF-α (**Q**) in CD45.1+ macrophages in LLC grafted tumors (*n*= 3 per group). Data are presented as mean ± SEM. ns: non-significant, * *p* < 0.05, ** *p* < 0.01, *** *p* < 0.001, **** *p* < 0.0001 by the *Student’s t* test (**A**-**Q**). Unless indicated, data were the representative of two independent experiments

We further analyzed macrophage profiles in the TME of subcutaneously grafted tumors from either PBS or BCG-treated mice. Our results showed that compared to the PBS control mice, BCG treatment significantly increased the percentages of tumor infiltrating mono-macrophages in LLC grafted tumors (Fig. 2A-C, Supplementary Fig. 4A). Tumor infiltrating mono-macrophages from BCG-trained mice displayed activated signatures including upregulated CD80, CD86 and MHC-II expression (Supplementary Fig. 4B-4D), whereas CD163 and CD206 (Supplementary Fig. 4E-4F) were dramatically down-regulated when compared to those from control tumors. Consistent with the characteristics of trained immunity, mono-macrophages from BCG-treated mice also produced higher levels of TNF-α and pro-IL1β (Supplementary Fig. 4G-4H).

Tumor infiltrating macrophages, also known as tumor-associated macrophages (TAMs) exhibit remarkable heterogeneity [31]. A recent work has classified TAMs into three subsets including TAM1 (CD64⁺CCR2⁺), TAM2 (CD73⁺), and TAM3 (CD11c⁺) [15]. In vitro flow cytometry on BCG-trained macrophages displayed their TAM1-like characteristics, marked by increased CD64 and CCR2 expression, and reduced CD73, CD11c and Ly6C expression (Fig. 2D). In LLC grafted tumors, BCG treatment enriched TAM1 while the proportions of TAM2/TAM3 as well as monocytes in LLC tumors were decreased when compared to those from control tumors (Fig. 2E and F). Notably, TAM1 from BCG treated tumors showed more production of TNF-α and pro-IL1β, while TAM2 and TAM3 remained unchanged as compared to those from control tumors (Supplementary Fig. 5A-5B).

Ms4a3 is identified as a biomarker to distinguish the origin of the macrophages either from *de novo* bone marrow (BM) hematopoiesis (Ms4a3^**+**^) or from tissue-resident macrophages generated from embryonic hematopoiesis (Ms4a3^**−**^) [32]. To trace the origin of TAMs in BCG-trained tumor-burden mice, we established BCG-trained anti-tumor models using Ms4a3^*Cre*^_Rosa^*tdTomato*^ reporter mice (Fig. 2G). It was found that BCG-trained Ms4a3^*Cre*^_Rosa^*tdTomato*^ mice suppressed tumor growth as well (Fig. 2H and I). The frequencies of Ms4a3^+^ mono-macrophages from BCG treated mice were significantly higher than those from PBS treated mice (Fig. 2J and K). More specifically, the proportions of Ms4a3^+^ mono-macrophages among CD45^+^ tumor-infiltrating immune cells were significantly elevated in BCG-trained mice as compared to those from PBS control mice whereas the proportions of Ms4a3^−^ mono-macrophages were comparable between BCG-trained and PBS group mice (Fig. 2L). Furthermore, we found that in Ms4a3^+^ TAMs the percentages of TAM1 were significantly increased in BCG-trained mice, but those of TAM2 and TAM3 remained unchanged between two group mice (Fig. 2M). Functionally, Ms4a3^+^ TAMs expressed higher levels of TNF-α and pro-IL1β after BCG training than Ms4a3^−^ counterparts (Supplementary Fig. 5C-5D).

To determine the effects of BCG-triggered BM hematopoiesis on the replenishment of tumor infiltrating TAMs, we also detected the expression of TAM1/2/3-like markers on monocytes in the BMs from either PBS or BCG treated mice (Supplementary Fig. 5E). We found that BCG treatment up-regulated CCR2 and CD64 expression in the monocytes residing in the BMs (Supplementary Fig. 5F-5G), while CD73 and CD11c remained unchanged (Supplementary Fig. 5H-5I). Furthermore, we sorted the monocytes from the BM of CD45.1 mice treated with PBS or BCG 7 days later, and intratumorally injected them into the grafted LLC tumors in CD45.2 mice at Day 13. The tumors were collected to detect the subtypes and functions of TAMs at Day 16 (Fig. 2N). The results showed that monocytes derived from the BM of BCG-trained mice differentiate into TAM1 and produced more IL-1β and TNF-α when entering the TME with more extent, indicating that BCG treatment reprogramed hematopoiesis leading to the differentiation of monocytes with high expression of CCR2 and CD64 facilitating their infiltration into the TME (Fig. 2O and Q).

To verify the involvement of CCR2 expression in promoting the infiltration of mono-macrophages in the TME, we firstly conducted an in vitro transwell assay that PBS or BCG trained macrophages were inoculated into the upper chamber, while LLC cells were inoculated into the lower chamber. We added DMSO or CCR2 inhibitor PF-4136309 and incubated the cells for 48 h. Afterwards, flow cytometry was used to detect the number of transferred macrophages in the lower chamber (Supplementary Fig. 6A). Our results showed that BCG-trained macrophages were more likely to migrate towards tumor cells, and the effects were significantly attenuated when CCR2 inhibitors were added (Supplementary Fig. 6B). For in vivo evidence, we intraperitoneally injected CCR2 inhibitors into LLC inoculated PBS or BCG trained mice (Supplementary Fig. 6C). We found that CCR2 inhibition significantly impaired BCG induced anti-tumor effects (Supplementary Fig. 6D-6E) and mono-macrophages infiltration within the TME (Supplementary Fig. 6F-6G). Moreover, the inhibition of CCR2 significantly decreased the generation of TAM1/2/3, especially CCR2^+^TAM1(Supplementary Fig. 6H–6J).

Therefore, BCG training induces BM hematopoiesis with more frequency of monocyte lineage development, conferring their capacity to differentiate into CD64^+^CCR2^+^ TAM1 subset and infiltrate into the TME exerting anti-tumor activity.

### ROS overproduction by TAMs dedicates to enhanced anti-tumor immunity in BCG-trained mice

**Fig. 3:**
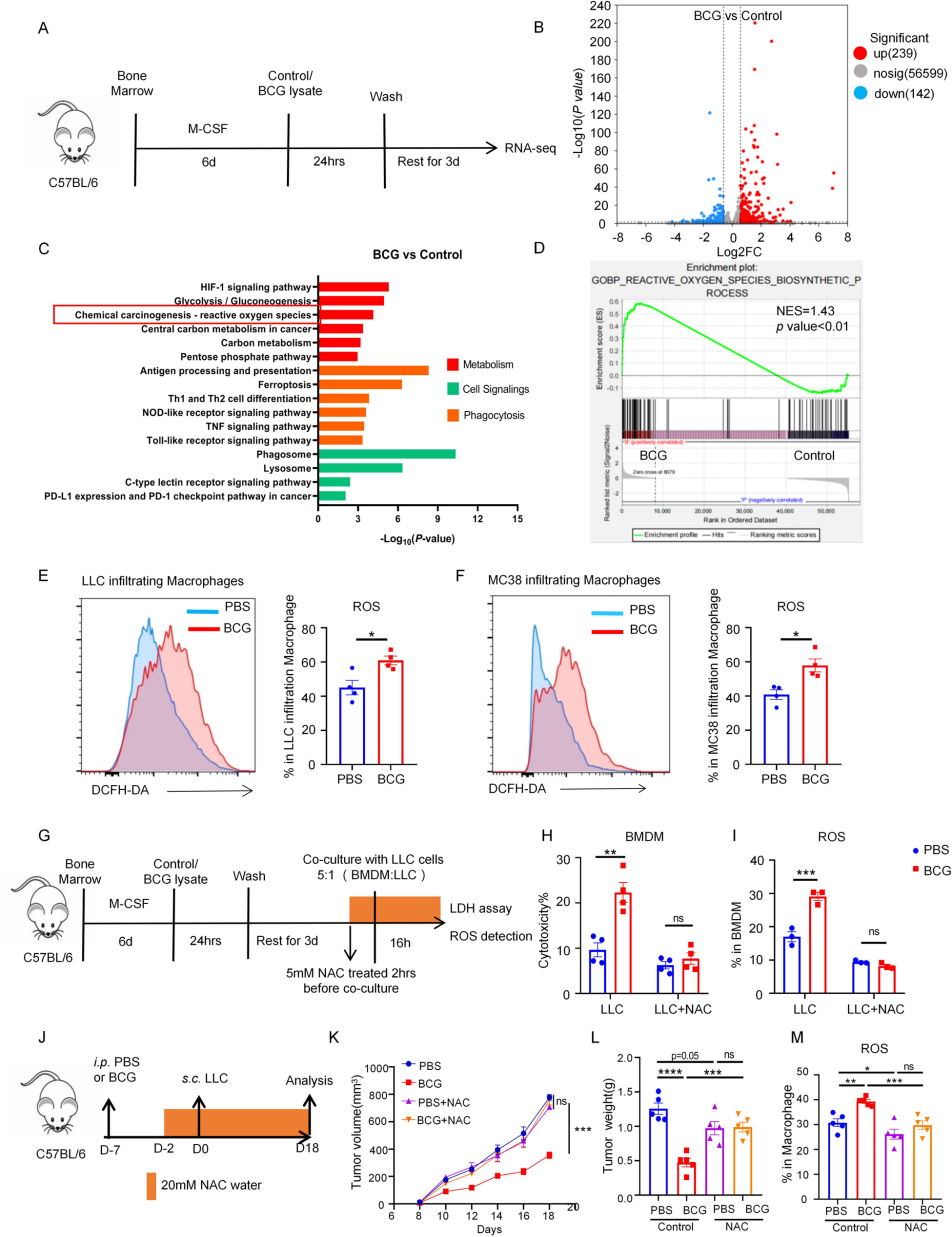
Increased ROS production in BCG-trained macrophages dedicates to enhanced anti-tumor immunity in BCG-trained mice. **A** Experimental scheme of transcriptome analysis of PBS and BCG-treated BMDMs. Briefly, BMDMs were induced by M-CSF for 6 days and treated with BCG lysates for 24 hrs. BMDMs was washed once with PBS, rested for 3 days and subject to RNA-sequencing. **B** Volcano plots indicating the alterations of gene expressing profiles in BCG-trained versus control BMDMs. **C** Enrichment of KEGG pathways upregulated in BCG-trained BMDMs as compared to control BMDMs. **D** GSEA analysis indicating the increase in ROS biosynthetic process in BCG-trained BMDMs as compared to control BMDMs. **E** Comparison of ROS levels (left) and ROS+ macrophages(right) in LLC grafted tumors from PBS and BCG-treated mice (*n*=4 per group). **F** Comparison of ROS levels (left) and ROS+ macrophages (right) in MC38 grafted tumors from PBS and BCG-treated mice (*n*=4 per group). **G** Experimental scheme of the effects of ROS production on in vitro cytotoxicity of PBS or BCG-treated BMDMs using LDH release assay. BMDMs were pre-treated with N-Acetyl-cysteine (NAC), a well-known antioxidant to neutralize ROS before the coculture with LLC cells. **H** Comparisons of in vitro cytotoxicity of BMDMs to LLC cells in PBS or BCG-trained BMDMs with or without the NAC treatment. Data were pooled from 4 individual mice. **I** Comparisons of the percentages of ROS-producing PBS or BCG-trained BMDMs with or without NAC treatment. Data were pooled from 3 individual mice. **J** Experimental scheme of the effects of ROS on in vivo inhibition of tumor growth by using the NAC inhibitor. **K**-**L** Comparisons of tumor growth curves (**K**) and tumor weight (**L**) of LLC grafted tumors in control and BCG-treated mice with or without NAC injection (*n*=5 per group). **M** Comparisons of ROS+ macrophage in LLC grafted tumors from PBS and BCG-treated mice with or without NAC injection (*n*=5 per group). Data are presented as mean ± SEM. ns: non-significant, * *p* < 0.05, ** *p* < 0.01, *** *p* < 0.001, **** *p* < 0.0001 by the Student’s t test (**E**-**F**) and One-way ANOVA (**H**-**M**). Unless indicated, data were the representative of two independent experiments

Having established the critical roles of macrophages in BCG-induced exaggerated anti-tumor immunity, we next investigated the underlying mechanisms via transcriptomic profiling. BMDMs were stimulated with BCG lysate or control medium for 24 h, washed, and rested for 3 days before bulk RNA sequencing (Fig. 3A). After BCG training, BMDMs significantly upregulated 239 genes and downregulated 142 genes (FC > 1.5, p value < 0.05) (Fig. 3B). KEGG pathway enrichment analysis in BCG-trained BMDMs revealed significant enrichment of pathways linked to metabolism process including reactive oxygen species (ROS) generation and glucose metabolism, cell signaling like antigen processing and presentation, and phagocytosis (Fig. 3C). The gene set enrichment analysis (GSEA) showed that genes related to ROS biosynthesis process were also significantly upregulated after BCG training (Fig. 3D). The roles of ROS in anti-tumor immunity have been widely investigated with controversial effects [23–25]. β-glucan has been reported to induce anti-tumor effects with the upregulation of ROS pathway without further mechanism exploration [26, 27]. In our study TAMs from BCG-trained mice have showed a significant increase in ROS levels in LLC (Fig. 3E) and MC38 grafted tumors (Fig. 3F). Similarly, elevated ROS production was observed in BCG trained BMDMs (Supplementary Fig. 7A-7B) and Raw264.7 (Supplementary Fig. 7C-7D) upon LPS re-stimulation in vitro, indicating that elevated ROS production constitutes an additional feature of BCG-trained immunity in the macrophages.

To determine whether ROS overproduction mediates enhanced anti-tumor immunity upon BCG training, we added a ROS scavenger N-acetylcysteine (NAC) [33] in BCG-trained BMDMs (Fig. 3G) and Raw264.7 (Supplementary Fig. 7E) and determined the cytotoxicity against tumor cells in an in vitro co-culture system. Our results showed that NAC neutralization abolished the enhanced cytotoxicity of BCG-trained macrophages against tumor cells (Fig. 3H and Supplementary Fig. 7F), which coincided with the abrogation of ROS production (Fig. 3I and Supplementary Fig. 7G). We also fed mice with the water containing 20 mM NAC or normal drinking water (Fig. 3J) starting at tumor inoculation. As expected, retarded tumor growth (Fig. 3K) and low tumor weight (Fig. 3L) were reversed in NAC-trained mice accompanied by less ROS production in TAMs to the similar levels in PBS-treated mice (Fig. 3M).

Therefore, extra ROS production by TAMs contributes to enhanced anti-tumor immunity in BCG-trained mice.

### NADPH oxidase 2 complex drives ROS production in BCG-trained macrophages

**Fig. 4:**
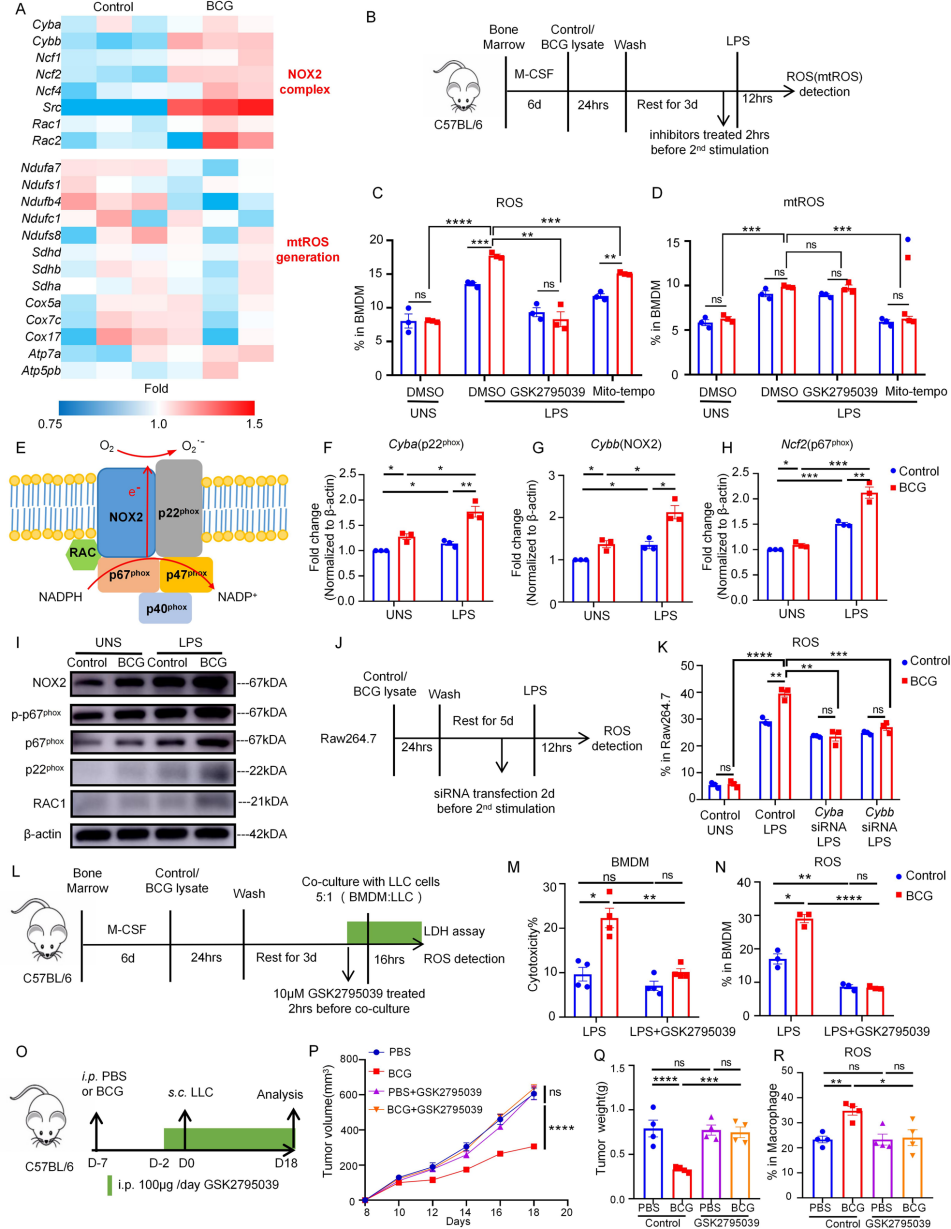
BCG-trained macrophages generate extra ROS in a NOX2 complex-dependent manner. **A** Heatmaps of gene expression levels involved in ROS generation PBS and BCG-trained BMDMs (*n* = 3). **B** Experimental scheme of ROS production in BCG-trained BMDMs upon LPS restimulation. **C**-**D** Comparisons of the percentages of ROS-positive (**C**) and mtROS-positive (**D**) BMDMs with control or BCG-training followed by LPS restimulation with or without GSK2795039 and mito-tempo treatment, two inhibitors to inhibit NOX2 oxidase activity and the production of mtROS, respectively. Data were pooled from 3 individual mice. **E** Structure model of NOX2 complex. **F**-**H** Expression levels of NOX2 complex components Cyba (**F**), Cybb (**G**) and Ncf2 (**H**) in control or BCG-trained BMDMs upon LPS treatment by quantitive-PCR. Data were pooled from 3 individual mice. **I** Immunoblotting of NOX2, *p*-p67^phox^(Thr233), p67^phox^, p22^phox^ and RAC1 in control or BCG-trained BMDMs upon LPS restimulation. **J** Experimental scheme of the effects of NOX2 complex expressions on ROS production in BCG-trained Raw264.7 upon LPS restimulation. **K** Percentages of ROS-producing Raw264.7 after control or BCG treatment followed by LPS restimulation with or without the suppression of *Cyba* and *Cybb* by siRNA interference. Data were the representative of three independent experiments. **L** Experimental scheme of in vitro cytotoxicity assay in BCG-trained BMDMs that was affected by NOX2 complex activity by the LDH release assay. **M** Comparisons of in vitro cytotoxicity of PBS and BCG-trained BMDMs against LLC cells with or without GSK2795039 treatment. Data were pooled from 4 individual mice. **N** Comparisons of the percentages of ROS-producing BMDMs with PBS or BCG treatment at the presence of vehicle and GSK2795039. Data were pooled from 3 individual mice. **O** Experimental scheme of in vivo anti-tumor effects of NOX2 complex activity in BCG-trained mice. **P**-**Q** Determination of tumor growth curves (**P**) and tumor weight (**Q**) of LLC grafted tumors in PBS and BCG-trained mice with or without the injection of GSK2795039 (*n*=4 per group). **R** Percentages of ROS-producing macrophages in LLC grafted tumors from PBS and BCG-trained mice with or without the injection of GSK2795039 (*n*=4 per group). Data are presented as mean ± SEM. ns: non-significant, * *p* < 0.05, ** *p* < 0.01, *** *p* < 0.001, **** *p* < 0.0001 by the One-way ANOVA (**F**-**G**, **M**-**R**) and two-way ANOVA with Holm-Sidak’s test (**C**, **D**, **K**). Unless indicated, data were the representative of two independent experiments

Intracellular ROS in the macrophages primarily originates from two pathways including cytoplasmic ROS (cROS) via the activation of NADPH oxidase and mitochondrial ROS (mtROS) [34]. When we re-analyzed the transcriptomic data of BCG-trained and control BMDMs, we found that the expression levels of genes related to NADPH oxidase 2 (NOX2) complex were significantly upregulated after BCG training, including *Cyba*,* Cybb*,* Ncf1*,* Ncf2 etc.*. However, there was no significant difference in gene expressions related to mtROS generation (Fig. 4A). To further dissect the contribution of NOX2 complex and mitochondria in extra ROS generation by BCG-trained macrophages, we treated BCG-trained macrophages with a NOX2 inhibitor GSK2795039 [35] and a mitochondrial scavenger Mito-tempo [36] upon in vitro LPS re-stimulation (Fig. 4B). We found that total ROS levels were elevated in BCG-trained BMDMs upon LPS re-stimulation (Fig. 4C) whereas mtROS levels were comparable between BCG and vehicle treated BMDMs (Fig. 4D). Addition of GSK2795039 abrogated ROS overproduction in BCG-trained BMDMs but had no effects on mtROS production. By contrast, Mito-tempo had no impact on either total ROS or mtROS pools after LPS stimulation (Fig. 4C and D). Transmission electron microscopy revealed no differences in mitochondrial length or abundance between BCG-trained and control BMDMs (Supplementary Fig. 8A-8 C). Therefore, our results indicate that the NOX2 complex rather than the mitochondria is the primary source of extra ROS in BCG-trained macrophages.

As illustrated in Fig. 4E, the NOX2 complex consists of multiple subunits that p22^phox^ and NOX2 subunits anchor in the cytosol membrane while p47^phox^, p67^phox^ and p40^phox^ attach to p22^phox^ and NOX2 exposing their binding sites to NADPH. With the assistance of RAC protein, NOX2 catalyzes O_2_ into O2**·-** a form of ROS with the conversion of NADPH into NADP^+^ [34, 37]. Accordingly, we detected the expression levels of the NOX2 complex key components by qPCR and Western blot in BCG-trained BMDMs. We found that the expressions of *Cyba*,* Cybb*,* Ncf2* genes **(**Fig. 4F and H**)** and their corresponding proteins p22^phox^, NOX2, p67^phox^ (Fig. 4I) were significantly upregulated in BCG-trained BMDMs as compared to control BMDMs after LPS re-stimulation. When we added siRNA to silence *Cyba* and *Cybb* in Raw264.7 (Fig. 4J), it was showed that silencing of both *Cyba* and *Cybb* significantly inhibited extra ROS generation in BCG-trained macrophages (Fig. 4K). These findings indicate that BCG-trained macrophages produce extra ROS in a NOX2 complex dependent manner.

We further investigated whether enhanced anti-tumor activity of BCG-trained macrophages were also related to the NOX2 complex. When we added GSK2795039 into BCG-trained BMDMs (Fig. 4L) or Raw264.7 (Supplementary Fig. 8D) and detected the cytotoxicity against LLC tumor cells in vitro, we found that the cytotoxicity of BCG-trained BMDMs and Raw264.7 was attenuated (Fig. 4M and Supplementary Fig. 8E) together with the reduction of ROS production (Fig. 4N and Supplementary Fig. 8F). Consistently, when we silenced *Cyba* and *Cybb* genes in BCG-trained or control Raw264.7 in vitro by siRNA (Supplementary Fig. 8G), the killing ability to tumor cells (Supplementary Fig. 8H) and the extra ROS generation (Supplementary Fig. 8I) were significantly impaired as well. After *i.p.* injection of BCG and PBS for 5 days, we started daily *i.p.* injection of GSK2795039 once every 2 days before subcutaneous tumor inoculation (Fig. 4O). Our results showed that GSK2795039 injection dramatically restored tumor growth in BCG-trained mice (Fig. 4P and Q). ROS overproduction in TAMs was attenuated with the treatment of GSK2795039 in BCG-trained mice as well (Fig. 4R). Moreover, we transfected PBS or BCG trained BMDMs with *Cybb*-targeted siRNA to specifically silence the *Cybb* expression, and then mixed with LLC tumor cells and co-inoculated into C57BL/6 mice (Supplementary Fig. 8J). The results showed that *Cybb* silence significantly inhibited the anti-tumor effect of BCG trained BMDM as well (Supplementary Fig. 8K-8L).

These results therefore indicate that enhanced anti-tumor activity of BCG-trained macrophages depends on the activation of NOX2 complex and the subsequent overproduction of cROS overproduction.

### Epigenetic rewiring of NOX2 complex in BCG-trained macrophages mediates enhanced anti-tumor activities

Epigenetic reprogramming is one of the key regulatory mechanisms in trained immunity of the macrophages, enabling heightened pro-inflammatory cytokine production upon re-stimulation [1, 17–19]. Previous study has shown that BCG injection intravenously was able to reprogram the differentiation of hematopoietic stem cells through epigenetic modification, thereby influencing peripheral monocytes and macrophages after the induction of trained immunity [30]. In our study *i.p.* injection of BCG after 7 days also increased the proportions of Sca-1^+^c-kit^+^ (LSK) hematopoietic stem cells in the BM (Supplementary Fig. 9A-9C) as compared to those from control mice. The proportions of myeloid progenitors including short-term hematopoietic stem cells (ST-HSC), multipotent progenitor cells (MPP) and granulocyte monocyte progenitors (GMPs) were also significantly expanded after BCG treatment (Supplementary Fig. 9D-9F). These data indicate that *i.p.* injection of BCG also promotes de novo BM hematopoiesis with biased differentiation to myeloid lineage.

**Fig. 5:**
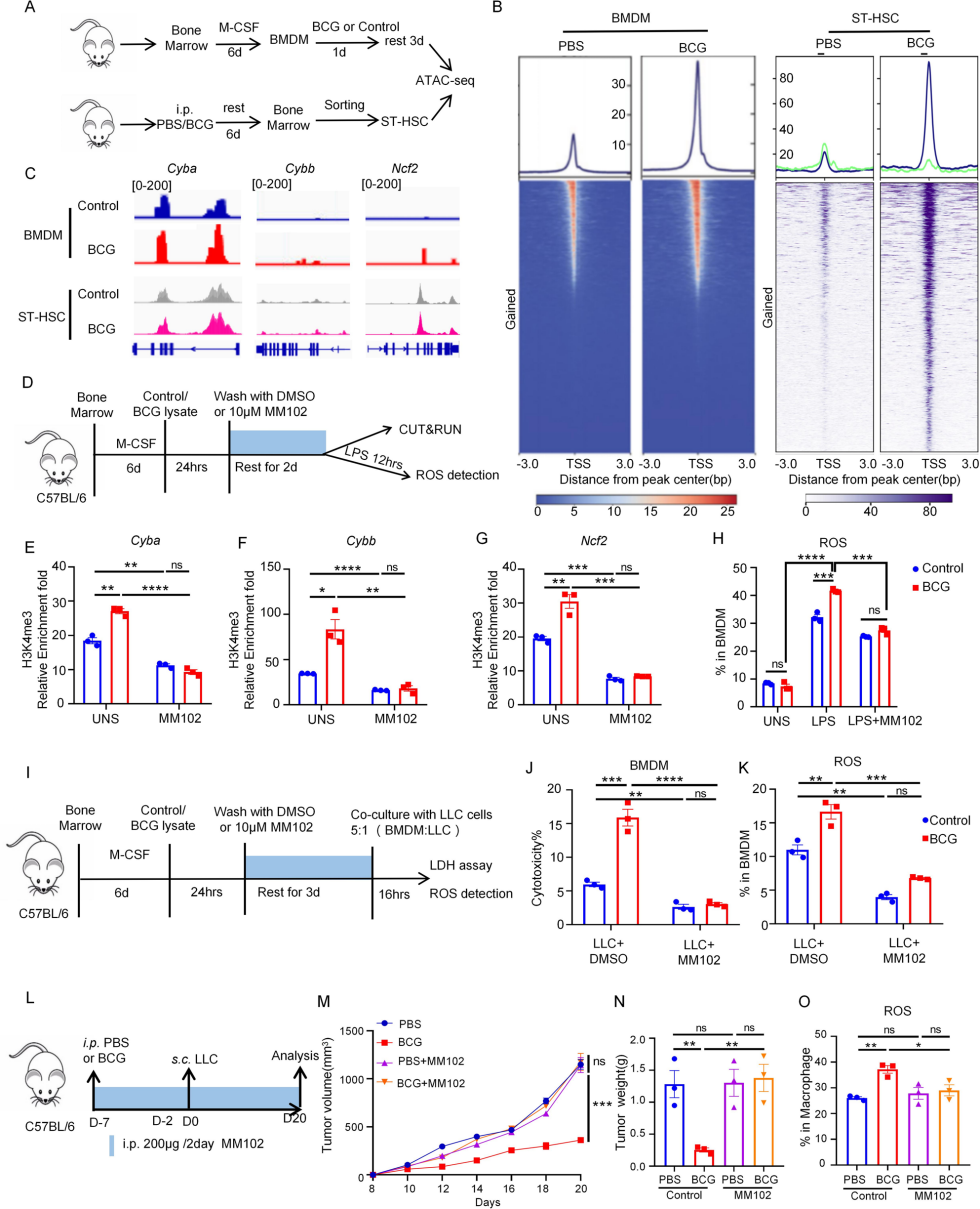
Epigenetic rewiring of NOX2 complex in BCG-trained macrophages mediates enhanced anti-tumor activities. **A** Experimental scheme of ATAC-seq analysis on PBS or BCG-trained BMDMs and bone marrow cells from PBS or BCG-treated mice. **B** Density plots and heatmaps of PBS or BCG-trained BMDMs and Short term- hematopoietic stem cells (ST-HSC) erased peaks by ATAC-seq analysis. **C** Integrative Genomics Viewer (IGV) track plots of chromatin accessibility for Cyba, Cybb and Ncf2 gene loci in PBS or BCG-trained BMDMs (upper) and ST-HSC (lower) from PBS and BCG-trained mice. **D** Experimental scheme of CUT&RUN analysis and ROS production in BCG-trained BMDMs upon LPS restimulation with or without the addition of a histone methyltransferase inhibitor MM102. **E**-**G** CUT&RUN analysis of H3K4me3 modification at the promoter regions of Cyba (**E**), Cybb (**F**) and Ncf2 (**G**) genes in PBS or BCG-trained BMDMs with or without MM102. The value was normalized to input control. Data were pooled from 3 individual mice. **H** Comparisons of ROS-producing control BMDMs and BCG-trained BMDMs upon LPS restimulation with or without the MM102 treatment. Data were pooled from 3 individual mice. **I** Experimental scheme of in vitro cytotoxicity assay in BCG-trained BMDMs that was affected by H3K4me3 modification activity by the LDH release assay. **J** Comparisons of in vitro cytotoxicity of PBS and BCG-trained BMDMs against LLC cells with or without MM102 treatment. Data were pooled from 3 individual mice. **K** Comparisons of the percentages of ROS-producing BMDMs with PBS or BCG treatment at the presence of vehicle and MM102. Data were pooled from 3 individual mice. **L** Experimental scheme of in vivo anti-tumor effects of H3K4me3 modification activity in BCG-trained mice. **M**-**N** Determination of tumor growth curves (**M**) and tumor weight (**N**) of LLC grafted tumors in PBS and BCG-trained mice with or without the injection of MM102 (*n*=4 per group). **O** Percentages of ROS-producing macrophages in LLC grafted tumors from PBS and BCG-trained mice with or without the injection of MM102 (*n*=4 per group). Data are presented as mean ± SEM. ns: non-significant, * *p* < 0.05, ** *p* < 0.01, *** *p* < 0.001, **** *p* < 0.0001 by the One-way ANOVA (**E**-**O**). Unless indicated, data were the representative of two independent experiments

Furthermore, we performed the assay for transposase-accessible chromatin with sequencing (ATAC-seq) assay on in vitro BCG-trained BMDMs and sorted ST-HSCs from the BM of in vivo BCG-trained mice to evaluate their chromatin openness (Fig. 5A). We found that both BMDMs and ST-HSCs showed a significant increase in chromatin openness after BCG training (Fig. 5B). The chromatin openness in the promoter regions of NOX2 complex related genes including *Cyba*,* Cybb and Ncf2* was also significantly upregulated in BMDMs and ST-HSCs after BCG training (Fig. 5C). Since BCG induced epigenetic reprogramming of the macrophages mainly manifested as an increase in the trimethylation of H3 histone 4 (H3K4me3) [2, 18, 30, 38], we conducted CUT&RUN experiments to detect the H3K4me3 levels at the promoter regions of NOX2 genes (Fig. 5D). Our results showed that after BCG training, H3K4me3 levels at the promoter regions of *Cyba* (Fig. 5E), *Cybb* (Fig. 5F) and *Ncf2* (Fig. 5G) were significantly upregulated. The upregulation of H3K4me3 may be attributed to either an increase in methyltransferases [39] or a decrease in demethylases [40]. Therefore, we assessed the transcript levels of methyltransferases and demethylases associated with H3K4me3 in RNA-seq data comparing BCG-trained BMDMs to control BMDMs. We found that genes related to WDR5 were significantly upregulated after BCG treatment (Supplementary Fig. 9G-9H), whereas there were no significant differences in the expression of demethylases such as *Kdm5a*, *Kdm5b*, *Kdm5c* and *Kdm5d* (Supplementary Fig. 9I-9L). We next conducted the experiments using MM102, an inhibitor of the MLL1/WDR5 methyltransferase [41]. Addition of MM102 completely abrogated BCG induced H3K4me3 methylation at these genes (Fig. 5E and G). Consistently, elevated levels of ROS in BCG-trained BMDMs were significantly suppressed when using MM102 upon LPS re-stimulation (Fig. 5H). Therefore, these results indicate that the H3K4me3 modification of NOX2 complex genes contributes to increased ROS generation in BCG-trained macrophages.

To determine the functional roles of H3K4me3 modification in BCG-trained macrophages with enhanced anti-tumor activity, we added MM102 into a BCG-trained BMDMs and LLC tumor cells co-culture system in vitro (Fig. 5I). MM102 treatment attenuated BCG-trained BMDM cytotoxicity (Fig. 5J) and reduced ROS production (Fig. 5K). We conducted *i.p.* injection of MM102 once every 2 days from BCG treatment and continued until the end of the tumor grafted experiments (Fig. 5L). The results showed that MM102 treatment significantly accelerated tumor growth in BCG-trained mice (Fig. 5M and N). ROS overproduction in TAMs from BCG-trained was also reduced to the similar levels of those from the control group mice upon MM102 treatment (Fig. 5O). Therefore, the enhanced anti-tumor effects on BCG-trained macrophages depends on H3K4me3 histone modification on the NOX2 complex.

### Activation of NOX2 complex in BCG-trained macrophages with enhanced anti-tumor activity relies on NF-κb transcriptional activity

**Fig. 6:**
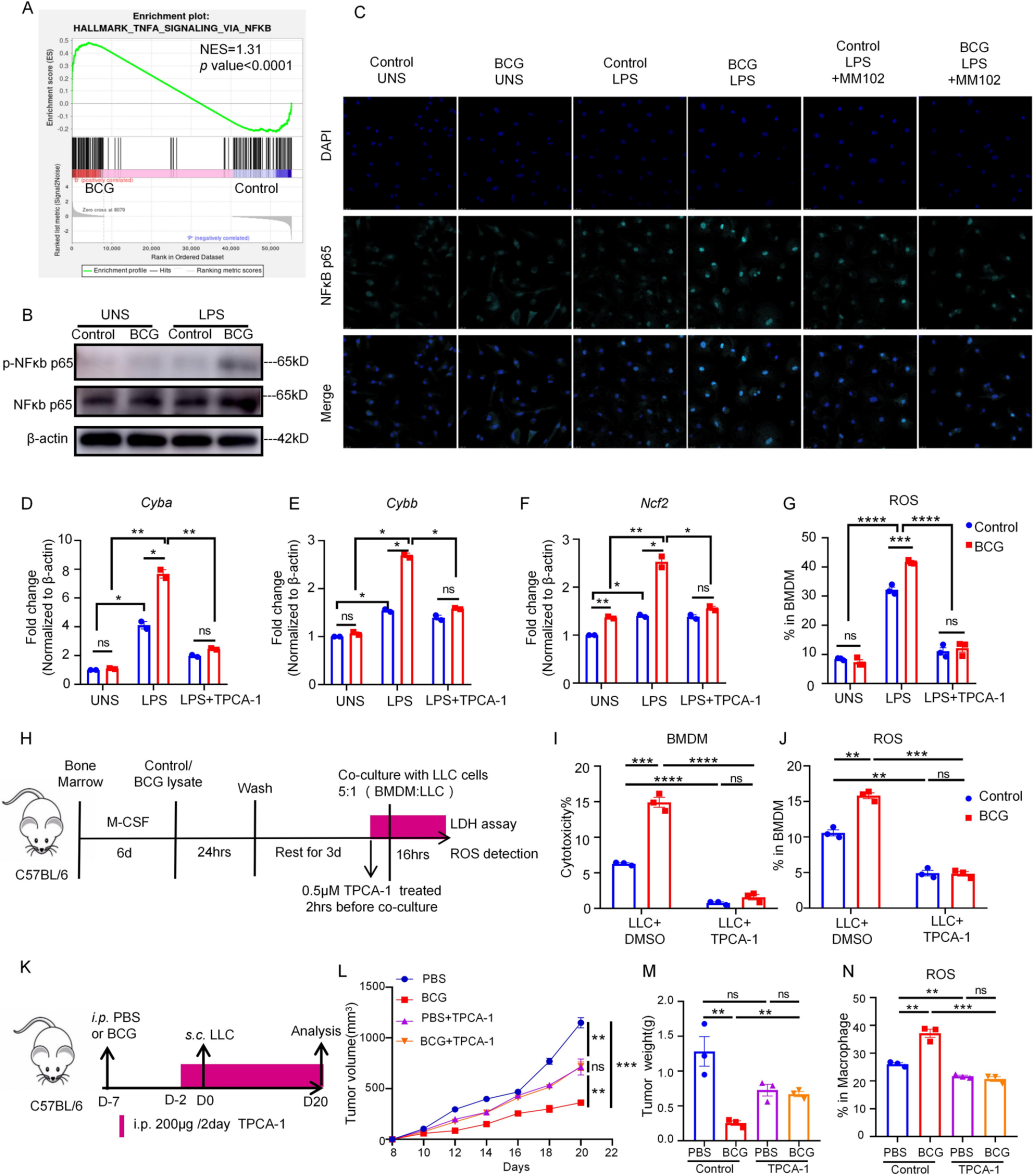
Activation of NOX2 complex in BCG-trained macrophages with enhanced anti-tumor activity relies on NF-κb transcriptional activity. **A** GSEA analysis indicating the activation of NF-κb signaling pathway in BCG-trained BMDMs as compared to control BMDMs. **B** Immunoblotting of p-NF-κb p65 (Ser536) and NF-κb p65 in control or BCG-trained BMDMs upon LPS restimulation. **C** Immunofluorescence staining of NF-κb p65 and DAPI in Control and BCG-trained BMDM with or without LPS re-stimulation and MM102 treatment. Green (Cy5) indicated NF-κb p65 and Blue (DAPI) indicated the nucleus. **D**-**F** Comparisons of *Cyba* (**D**), *Cybb* (**E**) and *Ncf2* (**F**) expression levels in control and BCG-trained BMDMs upon LPS restimulation with or without the NF-κb inhibitor TPCA-1 by qPCR. Data were pooled from 3 individual mice. **G** Comparisons of ROS-producing control BMDMs and BCG-trained BMDMs upon LPS restimulation with or without the NF-κb inhibitor TPCA-1. Data were pooled from 3 individual mice. **H** Experimental scheme of in vitro cytotoxicity assay in BCG-trained BMDMs that was affected by NF-κB activity by the LDH release assay. **I** Comparisons of in vitro cytotoxicity of PBS and BCG-trained BMDMs against LLC cells with or without TPCA-1 treatment. Data were pooled from 3 individual mice. **J** Comparisons of the percentages of ROS-producing BMDMs with PBS or BCG treatment at the presence of vehicle and TPCA-1. Data were pooled from 3 individual mice. **K** Experimental scheme of in vivo anti-tumor effects of NF-κB activity in BCG-trained mice. **L**-**M** Determination of tumor growth curves (**L**) and tumor weight (**M**) of LLC grafted tumors in PBS and BCG-trained mice with or without the injection of TPCA-1 (*n*=4 per group). **N** Percentages of ROS-producing macrophages in LLC grafted tumors from PBS and BCG-trained mice with or without the injection of TPCA-1 (*n*=4 per group). Data are presented as mean ± SEM. ns: non-significant, * *p* < 0.05, ** *p* < 0.01, *** *p* < 0.001, **** *p* < 0.0001 by the One-way ANOVA (**D**-**N**). Unless indicated, data were the representative of two independent experiments

Accumulating evidence demonstrates that the upregulation of H3K4me3 is beneficial for chromatin accessibility, which in turn enables transcription factors like NF-κB to bind promoter regions and drive gene transcription [42, 43]. Since the promoter sites of NOX2 complex genes, including *Cyba*,* Cybb* and *Ncf2*, have binding sites for NF-κB [44–46], chromatin opening of promoter regions in these genes might facilitate the binding of NF-κB to mediate the transcription of these genes. In fact, when we conducted GSEA analysis on the transcriptomic data of BCG-trained and control BMDMs, NF-κB related signaling pathway was significantly activated after BCG training (Fig. 6A). The phosphorylation level of NF-κB p65 in BCG-trained BMDMs was higher than that in control BMDMs after LPS re-stimulation (Fig. 6B). Nuclear localization of NF-κB p65 was dramatically increased in BCG-trained macrophages after LPS re-stimulation whereas attenuated after MM102 treatment (Fig. 6C). Furthermore, we used an NF-κB inhibitor TPCA-1 [47] to inhibit NF-κB activity. The upregulation of *Cyba*, *Cybb*, and *Ncf2* expressions was suppressed in BCG-trained macrophages (Fig. 6D and F) and excessive ROS generation was reduced (Fig. 6G). When we added TPCA-1 in BCG-trained BMDMs (Fig. 6H) that were co-cultured with tumor cells in vitro, the cytotoxicity to tumor cells of BCG-trained BMDMs has almost disappeared (Fig. 6I) with very low level of ROS production (Fig. 6J). After *i.p.* injection of BCG and PBS for 5 days we injected of TPCA-1 once every 2 days (*i.p.*) before subcutaneous tumor inoculation (Fig. 6K). TPCA-1 injection dramatically reversed BCG-mediated tumor growth inhibition (Fig. 6L and M). ROS overproduction in TAMs was also completely eliminated upon TPCA-1 treatment of in BCG-trained mice (Fig. 6N). Interestingly, we found that NF-κB inhibition alone could suppress tumor growth as well (Fig. 6L and M).

Collectively, these results indicate that the activation of NOX2 complex and enhanced anti-tumor immunity of BCG-trained macrophages relies on transcriptional activity of NF-κB.

### NOX2 complex gene signatures with trained immunity features predict favorable prognosis in BCG intravesical infusion therapy for non-muscle-invasive bladder cancers

**Fig. 7:**
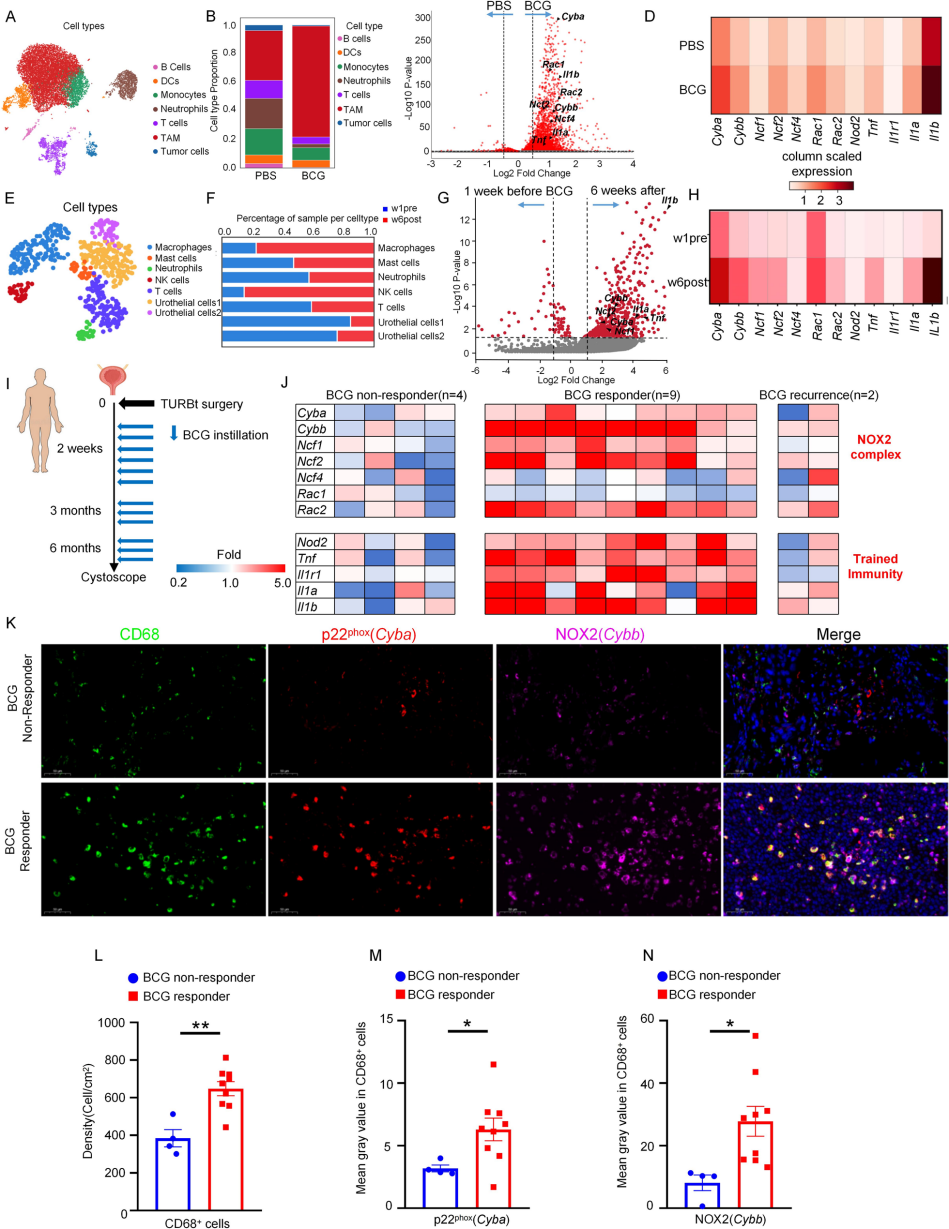
Upregulation of NOX2 complex genes in non-muscle-invasive bladder cancers with BCG-trained macrophage features correlates with favorable prognosis in BCG bladder infusion therapy. **A** UMAP dimensionality reduction plots for different cell types in bladder cancers from BCG treated group verse control group. **B** Percentage of different cell types in the bladder before BCG in the bladder of BCG treated group verse control group. **C** Volcano plots indicating the alterations of gene expressing profiles in the cells collected from the bladder in the bladder of BCG treated group verse control group. **D** Heatmaps showing relative expression of the genes involved in ROS generation by NOX2 complex and trained immunity signaling in the macrophages from the bladder of BCG treated group verse control group. **E** UMAP dimensionality reduction plots for different cell types in the urine before BCG treatment and after 6th BCG treatment. **F** Percentage of different cell types in the urine before BCG treatment and after 6th BCG treatment. **G** Volcano plots indicating the alterations of gene expressing profiles in the cells collected from the urine before BCG treatment and after 6^th^ BCG treatment. **H** Heatmaps showing relative expression of the genes involved in ROS generation by NOX2 complex and trained immunity signaling in the macrophages from the urine before BCG treatment and after 6th BCG treatment. **I** Therapeutic protocols of intravesical BCG instillation of non-muscle-invasive bladder cancer (NMIBC) patients. **J** Heatmaps showing relative gene expressions of NOX2 complex and trained immunity gene signatures in bladder cancers from BCG non-responder, (*n*=4) and BCG responder (*N*=9) NMIBC patients before receiving intravesical BCG instillation therapy and BCG recurrence (*n*=2) NMIBC patients after second cystoscope. **K** Representative immunofluorescence staining image of CD68(green), p22^phox^(red), NOX2(purple) and DAPI(blue) in bladder tumor of BCG non-responder (*n*=4) and BCG responder (*n*=9). **L** Comparisons of the density(cells per cm^2^) of CD68+cells in bladder tumor of BCG non-responder (*n*=4) and BCG responder (*n*=9). **M**-**N** Comparisons of the mean gray value of p22^phox^ (**M**) and NOX2 (**N**) in CD68+cells in bladder tumor of BCG non-responder (*n*=4) and BCG responder (*n*=9). Data are presented as mean ± SEM. * *p* < 0.05, ** *p* < 0.01 by the Student’s t test (**L**-**N**)

BCG intravesical instillation is the standard adjuvant therapy for non-muscle-invasive bladder cancer (NMIBC), achieving a clinical response rate of approximately 30% [48]. A recent study has reported that the effectiveness of BCG infusion therapy was associated with BCG-induced trained immunity which reprogrammed the hematopoiesis [49]. Thus, we reanalyzed the scRNA-seq data(GSE295309) of the tumors from mouse bladder with or without BCG treatment [49]. Through uniform manifold approximation and projection (UMAP) dimensionality reduction analysis, total cells from tumor samples were subdivided into 7 major subsets including B cells, DCs, monocytes, neutrophils, T cells, TAMs and tumor cells (Fig. 7A). The proportions of TAMs were significantly upregulated after BCG treatment whereas those of neutrophils, monocytes, T cells and tumor cells were downregulated (Fig. 7B). Gene expression volcano plot analysis on TAMs (Fig. 7C) revealed that the expressions of NOX2 complex-related genes including *Cyba*, *Cybb*, *Ncf1*, *Ncf2*, *Ncf4*, *Rac1* and *Rac2*, as well as trained immunity-related genes including *Nod2*, *Tnf*, *Il1r1*, *Il1a* and *Il1b* were significantly upregulated in BCG-treated group (Fig. 7D). We further classified the subtypes of TAMs using *Fcgr1* (encoding CD64) and *Ccr2 *for TAM1, *Nt5e* (encoding CD73) for TAM2, and *Itgax* (encoding CD11c) for TAM3, and found that the proportions of TAM1 significantly increased after BCG treatment, while the proportion of TAM2/3 significantly decreased (Supplementary Fig. 10A-10C). Furthermore, compared to TAM2/3, TAM1 exhibited higher expression levels of NOX2 complex-related genes and trained immunity related genes (Supplementary Fig. 10D-10E). These results indicates that BCG treatment promotes the polarization of macrophages within the BC TME toward the pro-inflammatory TAM1 phenotype.

In another study, Bhardwaj’s group collected urine-derived cells (UDCs) from BC patients one week before BCG instillation (w1pre) and 6 weeks after BCG instillation (w6post) to performed scRNA-seq analysis(GSE267718) [50]. Through UMAP analysis, UDCs were divided into urothelial cells1, urothelial cells2, T cells, macrophages, neutrophils, mast cells, and NK cells (Fig. 7E). We also observed the increase in the proportions of macrophages and neutrophils in the UDCs at w6post whereas those of T cells, NK cells and urothelial cells were downregulated (Fig. 7F). Gene expression volcano plot analysis on upregulated genes in w6post urine-derived macrophages showed the upregulation of NOX2 complex-related genes as well as trained immunity-related genes when compared to those at w1pre UDCs (Fig. 7G and H). These data provide additional evidence that BCG infusion induces macrophages with trained immunity features and hyperactivation of the NOX2 complex in clinical settings.

We next evaluated the clinical significance of NOX2 complex activation with respect to the prognosis in a small observational cohort including 13 NMIBC patients who receiving standard BCG infusion therapy. We have conducted the bulk-RNA sequencing on BC tumor samples before receiving BCG instillation. Among 13 BC patients, four were identified as the BCG non-responders with the recurrence within 6 months after BCG infusion therapy, and 9 as the BCG responders with no recurrence within 6 months. We also collected the paired tumor samples from 2 recurrent NMIBC patients (Fig. 7I and Supplementary Table 1). Interestingly, we have observed dramatic increases in NOX2 complex-related gene expressions including *Cyba*, *Cybb*, *Ncf1*, *Ncf2*, *Ncf4*, *Rac1*, *Rac2*, and trained immunity-related gene expressions including *Nod2*, *Tnf*, *Il1r1*, *Il1a*, *Il1b* in BCG responder-derived BC tumors before they received BCG infusion therapy when compared to those from non-responders and recurrent patients (Fig. 7J and Supplementary Fig. 10F-10G). We next performed immunofluorescence staining on tumor samples from BCG non-responders (*n* = 4) and BCG responders (*n* = 9) to determine macrophage infiltration and NOX2 complex expressions on the TAMs. Our results showed that the density of CD68^+^ macrophages in BC tumor samples from BCG responders was significantly higher than that from BCG non-responders (Fig. 7K and L). In addition, the mean fluorescence intensity (MFI) of p22^phox^ (Fig. 7M) and NOX2 (Fig. 7N) in CD68^+^ macrophages in tumor samples from BCG responders was also significantly higher than those from BCG non-responders. Collectively, these data provide preliminary clinical evidence that upregulation of NOX2 complex genes and trained immunity-featured genes might correlate with favorable outcomes in NMIBC patients who receiving BCG intravesical therapy.

## Discussion

Accumulating evidence highlights the critical roles of innate immune cells including macrophages [51], neutrophils [52] and NK cells [53] in dictating tumor initiation and progression. Given the plasticity of TAMs within the TMEs [54], strategies to polarize TAMs toward anti-tumor characters represent a promising avenue for both preventing tumor progression and enhancing cancer immunotherapy. In this study, we demonstrate that BCG-induced trained immunity confers pan-anti-tumor activity by promoting tumor infiltrating TAMs to produce elevated levels of ROS. Mechanistically, BCG training induces epigenetic remodeling in the macrophages with increased chromatin openness at NOX2 complex gene loci. This remodeling facilitates the accessibility of activated NF-κB to promote the transcription of NOX2 complex and subsequently robust ROS generation. BCG training before tumor inoculation polarizes the macrophages to an anti-tumor TAM1 phenotype. Upon tumor initiation, these trained macrophages preferentially migrate to the TME and exert enhanced anti-tumor immunity relying on excessive ROS production. Clinically, NMIBC patients with elevated NOX2 complex gene signatures in tumors exhibit a better prognosis of intravesical BCG treatment. Our study therefore provides compelling evidence that arming TME-resident macrophages with NOX2-dependent ROS production through BCG training represents a novel strategy to potentiate cancer immunotherapy.

Given the critical role of macrophage plasticity in the TME dynamics and immunotherapy outcomes, we have firstly investigated the origin of TAMs following BCG training. Previous studies show that tissue macrophages are derived from either embryonic precursors or adult circulating monocytes with tissue-specific proportions of these two ontogenically distinct populations [55]. In the TME, macrophages are the most dominant and early infiltrating immune cells [56]. For instance, nearly 70% of TAMs are monocyte-derived and exhibit different functional profiles from embryonically derived TAMs in a murine model of PDAC [57]. Differential localization of embryonic and monocyte-derived TAMs was also observed in murine and human lung cancers [58, 59]. Our study, along with others, demonstrates that BCG training acts at the level of hematopoietic stem cells (HSCs) in the bone marrow (BM). Leveraging a myeloid lineage-tracking system with *Ms4a3* as a specific marker for BM-derived monocytes, we demonstrate that BCG training promotes *de novo* BM hematopoiesis. This process generates TAMs that are predominantly derived from circulating monocytes in BCG-trained mice. BCG-treated monocytes acquired trained immunity features as well as upregulated chemokine receptors (e.g., CCR2) to facilitate tumor infiltration [60–62]. Notably, tumors from PBS and BCG-trained mice contained comparable proportions of *Ms4a3*-negative macrophages (putative embryonic precursors) with similar pro-IL-1β and TNF-*α* production (Supplementary Fig. 5C-5D). Although the precise origin of *Ms4a3*-negative macrophages remains unclear, our data indicate that BCG-induced enhanced anti-tumor activity primarily arises from mobilization of BM hematopoiesis. In contrast, embryonically derived peripheral macrophages exhibit a substantially blunted responsiveness to BCG training. This is likely due to their terminally differentiated stage restricting functional reprogramming. The underlying molecular mechanisms governing this phenomenon warrant further investigation.

Interestingly, we also observed that 80% of the TAMs in PBS-treated mice were *Ms4a3*-positive whereas tumor burden in non-BCG trained mice was higher than that from BCG trained mice. This is likely because BCG training has driven more early myeloid progenitors to differentiate into tumor-suppressive myeloid cells before tumor burden. Moreover, a recent study has classified the TAMs into TAM1, TAM2 and TAM3 among which TAM2 and TAM3 exhibit pro-tumor activity [15]. In our study, the percentages of TAM2 and TAM3 were comparable between BCG and PBS-treated mice. However, TAM1 subpopulation was enriched in the tumor regions from BCG-trained mice, which is consistent with more tumor suppression. Cytokine profiling revealed that only TAM1 secreted elevated IL-1β and TMF-α, two hallmark cytokines of trained immunity. Trajectory analysis showed that monocytes differentiate into TAM1 transient intermediate before maturing into terminally differentiated TAM2/TAM3 [15]. BCG treatment preferentially promoted monocyte-to-TAM1 differentiation without affecting terminal TAM1/TAM2 differentiation. Given TAM1 gene expression profiles associated with chemotaxis together with trained immunity properties, they presumably migrate more effectively to tumor sites upon BCG training. The increases in *Ms4a3*^+^ macrophages in the tumor regions from BCG-trained mice is consistent with these results. On the contrary, non-BCG trained mice showed preferential development of pro-tumorigenic myeloid cells where IL-4 signaling in the early myeloid progenitors in BM programmed the development of immunosuppressive tumor-promoting myeloid cells and led to tumor burden [63].

In this study, we further elucidated the critical role of excessive ROS production by TAMs in mediating BCG training induced enhanced anti-tumor immunity. While trained immunity was initially identified by overproduction of pro-inflammatory cytokines such as IL-1β, TNF-α, IL-6 upon restimulation [1, 2, 16], our recent work showed that BCG-trained macrophages also overproduced type I interferons during viral reinfection and augmented antiviral immunity [64]. Here, we likely identified ROS as another indicative molecule of BCG-induced trained immunity, expanding the known functional readouts of this phenomenon. In the anti-tumor scenario, clearance of ROS by NAC had a significant impact on the proportions and anti-tumor effects of the TAMs, as well as the decrease in CD80, CD86, pro-IL1β and TNF-α expression (Supplementary Fig. 7H-7 L). What needs to be emphasized is that the infiltration of BCG-trained macrophages in tumor regions could potentiate T cell functionality. Depletion of macrophages via using anti-F4/80 treatment impaired the functionality of tumor infiltrating CD8^+^ T cells, as evidenced by significantly decreased cytokine production and cytotoxicity whereas the proportions were comparable (Supplementary Fig. 1I-1 L). In addition, ROS clearance also significantly decreased CD8^+^T cells infiltration as well as the production of IFN-γ, TNF-α, CD107A, and Granzyme B and CD8^+^T cells (Supplementary Fig. 7M-7Q). These findings in part imply a functional association between BCG-induced TAMs and T cell immunity, which is also in line with a recent study on scRNA-Seq analysis on the monocyte expression profiles from bladder cancer patients before and after receiving BCG intravesical instillation treatment [50].

The expansion of BCG-trained immunity repertoire is linked to epigenetic remodeling in the macrophages undergoing trained immunity. The ATAC-seq results from ours and other groups have demonstrated global chromatin accessibility related to multiple biological processes [65], including cytokine and ROS production. One of the interesting findings in our study is that BCG-trained TAMs generate ROS predominantly via the NOX2 complex, one of the downstream pathways relying on NF-κb signaling activity. According to the previous studies, there are two major sources of intracellular ROS as NADPH oxidase dependent and mitochondria dependent production [34]. Our results showed that ROS overproduction in BCG-trained macrophages mainly depended on NOX2 complex rather than mitochondria sources (Fig. 4B and D). However, Ding’s group reported that the increased ROS generation in lung interstitial macrophages after tumor cell re-stimulation was mostly dependent on mtROS upon β-glucan training [26]. Kalafati’s work also showed mtROS dependency in β-glucan-trained neutrophil cytotoxicity [27]. Nevertheless, in our study transcriptomic analysis of BCG-trained BMDMs revealed no significant changes in mtROS-related gene expression. This might be due to the difference of two trained immunity inducers in the recognition (BCG by NOD2 and β-glucan by Dectin-1) [2, 66], and downstream signaling pathways. Elucidating pathway-specific mechanisms needs further investigation.

BCG is initially developed as an anti-TB vaccine and has been applied in anti-tumor scenario for more than 80 years [4, 67, 68]. While subcutaneous inoculation of BCG had minimal impact on BM hematopoiesis and is less effective in inducing long-term innate immune memory, intravenous BCG administration can elicit durable trained immunity with high safety risk [30]. Notably, viable BCG bacilli can persist in the bone marrow and other organs for up to 120 days after intravenous BCG injection in mice. Here we used intraperitoneal injection, and demonstrated that after 30 days BCG was undetectable in peritoneal lavage fluid whereas de novo BM hematopoiesis was maintained (data not shown), suggesting that *i.p.* BCG injection represents a moderate way to balance the anti-tumor efficacy and bio-safety risk. In clinic, intravesical BCG instillation is the standard therapy for preventing recurrence in NMIBC, but achieves a response rate of only ~ 30% [48]. How to increase the clinical efficacy of this preventive therapy is still lack of the guidance. Based on our study, we provide the preliminary hint that high expressions of trained immunity-related genes (including those governing ROS production) may be helpful to facilitate the stratification of bladder cancer patients with favorable outcome once validated in a large cohort study in the future, thereby providing a foundation for precision medicine in BCG-based immunotherapy.

## Conclusions

Our study has systematically investigated the mechanisms by which BCG-induced trained macrophages mediate anti-tumor immunity. BCG-treated macrophages exert favorable anti-tumor efficacy in tumor regions through increasing their tumor chemotaxis and ROS production. Epigenetically, the chromatin opening after BCG treatment is more conducive to the binding of transcription factors such as NF-κB to the promoter regions of functional genes, thereby promoting the expression of the NOX2 complex and ROS production. Clinically, high expressions of NOX2 and trained immunity-related genes are associated with better responses to BCG intravesical therapy in bladder cancer patients. Based on our comprehensive mouse experiments and clinical correlation analysis, we conclude that BCG training drives the macrophages to acquire key features including excessive ROS production and tumor-directed chemotaxis to potently exhibit anti-tumor immunity. This mechanism also offers a novel theoretical foundation for future clinical application of NCG in pan-anti-tumor therapies beyond bladder cancer.

### Limitation of the study

This study has several limitations. Firstly, the precise mechanisms by which cytosol ROS rather than mtROS that contributes to enhanced anti-tumor immunity in BCG-trained macrophages warrant further investigation. Second, we employed live BCG for in vivo induction of trained immunity and BCG lysate for in vitro induction. Given the complexity of BCG components and future clinical translation, to identify a single BCG-derived component as the inducer of trained immunity will be more feasible for their application in the future. This will also be more practical for elucidating the precise mechanisms underlying receptor-triggered downstream signal pathways involved in trained immunity. Thirdly, the duration of BCG-trained anti-tumor immunity and its cross-talk with adaptive immune system require in-depth exploration. 

## Supplementary Information

Supplementary Material 1. Supplementary Fig S1 related to Figure 1. BCG-trained mice exhibits enhanced TNF-α and IL-1β production and long period of anti-tumor effects. (A) Experimental scheme of TNF-α and pro-IL1β production of macrophage in spleen in PBS or BCG treated mice with or without LPS re-stimulation. (B-C) Comparison of TNF-α^+^(B) and pro-IL1β^+^(C) macrophage in spleen in PBS or BCG treated mice with or without LPS re-stimulation(*n*=5 per group). Data from two independent experiments were pooled and presented. (D) Experimental scheme. Briefly, BCG was intraperitoneally injected once and multiple subcutaneous grafted tumors were established after 6 weeks. Tumor growth curves were monitored and mice were sacrificed according to tumor volumes. (E-F) Tumor growth curves of grafted Lewis lung carcinoma (LLC) (E) and tumor weight (F) in PBS (*n* = 4) versus BCG treated (*n* =4) mice. (G) CD8^+^ T cells gating strategy. (H) Representative contour map and comparisons of the percentages of CD8^+^ T cells in CD45^+^ cells in LLC grafted tumors between PBS and BCG-treated mice with or without anti-F4/80 mAb treatment. (I-L) Representative contour map and comparisons of IFN-γ^+^(I), TNF-α^+^(J), CD107A^+^(K) and Granzyme B^+^(L) CD8^+^ T cells in LLC grafted tumors between PBS and BCG-treated mice with or without anti-F4/80 mAb treatment. Data are presented as mean ± SEM. ns: non-significant, * *p* < 0.05, ** *p* < 0.01, *** *p* < 0.001, **** *p* < 0.0001 by the Student’s t test (E-F) and one-way ANOVA (B,C,H-L). Supplementary Fig S2 related to Fig. 1. BCG-trained mice exhibit enhanced anti-tumor effects depending on BCG-trained macrophages. (A-D) Experimental scheme of anti-tumor immunity in PBS or BCG-trained WT or nude mice. Briefly, PBS or BCG was injected once and mouse colorectal cell line MC38 was inoculated in C57BL/6 and nude mice (A). Tumor growth curves (B) were monitored every 2 days. Tumor weight (C) and the percentage of CD8^+^T cells (D) were assayed at Day 16 after the sacrifice of PBS and BCG-trained C57BL/6 and nude mice(*n*=4 per group). (E-H) Experimental scheme of anti-tumor immunity in BCG-trained mice with the depletion of CD8+T cells. Briefly, BCG was injected once and mouse LLC cell lines was inoculated in C57BL/6 mice at Day 7. Anti-mouse CD8 antibodies were injected one day before LLC inoculation (Day -1) and Day 6, 13 (E). Tumor growth curves (F) were monitored every 2 days.Tumor weight (G) and the percentage of CD8+T cells(H) were assayed at Day 18 after the sacrifice of PBS and BCG-trained mice with or without anti-CD8 mAb treatment(*n*=3 per group). (I-L) Experimental scheme of anti-tumor immunity in BCG-trained mice with the depletion of NK cells. Briefly, BCG was injected once and mouse LLC cell lines was inoculated in C57BL/6 mice at Day 7. Anti-mouse NK1.1 antibodies were injected one day before LLC inoculation (Day -1) and Day 4, 9, 12 (I). Tumor growth curves (J) were monitored every 2 days.Tumor weight (K) and the percentage of NK cells(L) were assayed at Day 18 after the sacrifice of PBS and BCG-trained mice with or without anti-NK1.1 mAb treatment (*n*=3 per group). (M-P) Experimental scheme of anti-tumor immunity in BCG-trained mice with the depletion of neutrophils. Briefly, BCG was injected once and mouse LLC cell lines was inoculated in C57BL/6 mice at Day 7. Anti-mouse Ly6G antibodies were injected one day before LLC inoculation (Day -1) and Day 2, 5, 8, 10 and 15 (M). Tumor growth curves (N) were monitored every 2 days.Tumor weight (O) and the percentage of neutrophils (P) were assayed at Day 18 after the sacrifice of PBS and BCG-trained mice with or without anti-Ly6G mAb treatment (*n*=4 per group). Data are presented as mean ± SEM. ns: non-significant, * *p* < 0.05, ** *p* < 0.01, *** *p* < 0.001, **** *p* < 0.0001 by one-way ANOVA (B-P). Supplementary Fig S3 related to Figure 1. BCG-trained macrophages exhibits enhanced TNF-α and IL-1β production after LPS re-stimulation and higher cytotoxicity to LLC and MC38 cells. (A) Experimental scheme of TNF-α and pro-IL1β production of bone marrow derived macropage(BMDM) control or BCG-trained BMDM with or without LPS re-stimulation. (B-C) Comparison of TNF-α(B) and IL1β (C) production in BMDM control or BCG-trained BMDM with or without LPS re-stimulation. Data were pooled from 3 individual mice. (D) Experimental scheme of in vitro cytotoxicity of BCG-treated bone marrow derived macrophages (BMDMs) against tumor cells. Briefly, BMDMs were induced by M-CSF for 6 days and treated with BCG lysates for 24 hrs. BMDMs were washed once with PBS and rested for 3 days. Then BMDMs were incubated with tumor cell lines at 5:1 ratios of effector to target cells. The culture supernatants were collected 4 hrs later and subjected to determine the LDH level according to the manufacturer’s instructions. (E) Comparison of the cytotoxicity of BMDMs to LLC and MC38 tumor cells with or without BCG treatment. Data were pooled from 4 individual mice and presented. (F) Experimental scheme of TNF-α and pro-IL1β production of control or BCG-trained Raw264.7 cells with or without LPS re-stimulation. (G-H) Comparison of TNF-α (G) and IL1β(H) production in control or BCG-trained Raw264.7 with or without LPS re-stimulation. (I) Experimental scheme of the effects on in vitro cytotoxicity of control or BCG-treated RAW264.7 cells using LDH release assay. (J) Comparisons of in vitro cytotoxicity of control or BCG-trained RAW264.7 cells to LLC cells or MC38 cells. Data are presented as mean ± SEM. ns: non-significant, * *p* < 0.05, ** *p* < 0.01, *** *p* < 0.001, **** *p* < 0.0001 by the Student’s t test (E and J) and one-way ANOVA (B,C,G and H). Supplementary Fig S4 related to Fig. 2. BCG-trained mo-macrophages exhibits more activated characteristics. (A) Gating strategy. (B-H) Representative flow contour maps and comparisons of CD80^+^(B), CD80^+^(C), MHC-II^+^(D), CD163^+^(E), CD206^+^(F), pro-IL1β^+^(G) and TNF-α^+^(H) mono-macrophages in LLC grafted tumors between PBS and BCG-treated mice. Data are presented as mean ± SEM. * *p* < 0.05, ** *p* < 0.01, *** *p* < 0.001 by the Student’s t test (B-H). Supplementary Fig S5 related to Fig. 2. BCG induced increased production of TNF-α and pro-IL1β macrophages more produced by TAM1 and bone marrow derived macrophages. (A-B) Representative flow contour maps and comparisons of the percentages of pro-IL1β^+^(A) and TNF-α^+^(B)TAMs in TAM subsets in LLC grafted tumors between PBS (*n*=3) and BCG-treated mice (*n*=3). (C-D) Representative flow contour maps and comparisons of the percentages of pro-IL1β^+^ (C) and TNF-α^+^(D)TAMs in Ms4a3^+^ or Ms4a3^-^ macrophages in LLC grafted tumors between PBS (*n*=3) and BCG-treated mice (*n*=3). (E) Experiment scheme. (F-I) Comparisons of the percentages of CCR2^+^ (F), CD64^+^ (G), CD11c^+^ (H) and CD73^+^ (I) monocytes in bone marrow between PBS (*n*=3) and BCG-treated mice (*n*=3). Data are presented as mean ± SEM.ns: non-significant,* *p* < 0.05,** *p* < 0.01, *** *p* < 0.001 by the *Student’s t* test (A-I). Supplementary Fig S6 related to Figure 2. CCR2 drives BCG trained macrophages tumor infiltration and TAM1 generation. (A) Experimental scheme of BMDM transfer capacity assay by transwell system. Briefly, BMDMs were trained with PBS or BCG lysate for 24 hours. After wash the supernatant and rest for 2 days, 50000 BMDMs were inoculated on the upper chamber and 25000 LLC cells were inoculated on the lower chamber. The cells were treated with DMSO or 1μM CCR2 inhibitor PF-4136309 for 48 hours. The cells in lower chamber were digested and flow cytometry were used to determine the number of transferred BMDMs. (B) Comparison of the number of transferred BMDMs in lower chamber in the transwell system. Data were pooled from 3 individual mice. (C) Experimental scheme of in vivo anti-tumor effects in PBS or BCG-trained mice with or without CCR2 inhibitor PF-4136309. (D-E) Determination of tumor growth curves (D) and tumor weight (E) of LLC grafted tumors in PBS and BCG-trained mice with or without the injection of PF-4136309 (*n*=3 per group). (F-J) Percentages of Monocytes (F), TAMs (G), TAM1 (H), TAM2 (I) and TAM3 (J) in CD45^+^ cells in LLC grafted tumors from PBS and BCG-trained mice with or without the injection of PF-4136309 (*n*=3 per group). Data are presented as mean ± SEM. ns: non-significant, * *p* < 0.05, ** *p* < 0.01, *** *p* < 0.001, **** *p* < 0.0001 by the One-way ANOVA (B-J). Supplementary Fig S7 related to Figure 3. BCG-trained macrophages exhibit enhanced anti-tumor cytotoxicity relying on ROS production. (A,C) Experimental scheme of ROS detection between control and BCG-trained BMDM (A) or Raw264.7 cells (C) upon LPS re-stimulation. (B,D) Comparison of the percentage of ROS-producing BMDMs (B) or Raw264.7 cells (D) with or without BCG treatment upon LPS re-stimulation. (E) Experimental scheme of the effects on in vitro cytotoxicity of control or BCG-treated RAW264.7 cells using LDH release assay and ROS detection. (F) Comparisons of in vitro cytotoxicity of control or BCG-trained RAW264.7 cells to LLC cells with or without NAC treatment. (G) Comparisons of the percentages of ROS production in control or BCG-trained RAW264.7 cells with or without NAC treatment. (H) Comparisons of the percentages of macrophages in CD45^*+*^ cells in LLC grafted tumors between PBS and BCG-treated mice with or without NAC treatment (*n*=4 per group). (I-L) Comparisons of CD80^+^(I), CD86^+^(J), pro-IL1β^+^(K) and TNF-α^+^(L) macrophages in LLC grafted tumors between PBS and BCG-treated mice with or without NAC treatment. (M) Comparisons of the percentages of CD8+ T cells in CD45^+^ cells in LLC grafted tumors between PBS and BCG-treated mice with or without NAC treatment (*n*=4 per group). (N-Q) Comparisons of IFN-γ^+^(N), TNF-α^+^(O), CD107A^+^(P) and Granzyme B^+^(Q) CD8^+^ T cells in LLC grafted tumors between PBS and BCG-treated mice with or without NAC treatment (*n*=4 per group). Data are presented as mean ± SEM. ns: non-significant, * *p* < 0.05, ** *p* < 0.01, *** *p* < 0.001, **** *p* < 0.0001 by the One-way ANOVA (B-Q). Supplementary Fig S8 related to Fig. 4. BCG-trained BMDMs maintain normal mitochondrial structure and BCG-trained RAW264.7 generate extra ROS in a NOX2 complex-dependent manner. (A) Mitochondrial structures of BMDMs with or without BCG treatment by transmission electron microscopy. (B-C) Comparison of mitochondrial length (B) and numbers (C) between control and BCG-trained BMDMs. (D) Experimental scheme of in vitro cytotoxicity assay in BCG-trained RAW264.7 that was affected by NOX2 oxidase activity by the LDH release assay. (E) Comparisons of in vitro cytotoxicity of PBS and BCG-trained RAW264.7 to LLC cells with or without GSK2795039 treating. (F) Comparisons of the percentages of ROS-producing RAW264.7 cells with PBS or BCG treatment at the presence of vehicle and GSK2795039. (G) Experimental scheme of in vitro cytotoxicity assay in BCG-trained Raw264.7 that was affected by NOX2 complex gene expression through the LDH release assay. (H) Comparisons of in vitro cytotoxicity of PBS and BCG-trained Raw264.7 against LLC cells with or without the interference of *Cyba* and *Cybb* expressions (*n* = 3). (I) Comparisons of the percentages of ROS-producing Raw264.7 with or without the interference of *Cyba* and *Cybb* expressions (*n* = 3). (J) Experimental scheme of co-transferring of LLC tumor cells and PBS/BCG-trained BMDMs with mock or *Cybb* siRNA transfection in C57BL/6 mice (*n* = 4 per group). (K-L) Comparisons of tumor growth curves (K) and tumor weight (L) of mice inoculated by LLC cells mixed with PBS/BCG-trained BMDM with mock or Cybb siRNA transfection (*n* = 4 per group). Data are presented as mean ± SEM. ns: non-significant, * *p* < 0.05, ** *p* < 0.01, *** *p* < 0.001, **** *p* < 0.0001 by the Student’s t test (B-C) and One-way ANOVA (E-L). Supplementary Fig S9 related to Figure 5. Increased proportion of bone marrow hematopoietic stem cells in mice treated with BCG. (A-B) Representative flow contour maps (A) and quantifications (B) of the percentages of Lineage-Sca-1+c-Kit+(LSK) cells in lineage-bone marrow cells of PBS or BCG treated mice. (*n*=10 per group). Data from two independent experiments were pooled and presented. (C-E) Comparisons of the percentage of LSK (C), multi-potent progenitor (MPP)(D), short-term hematopoietic stem cells(ST-HSC)(E) in total bone marrow cells of PBS or BCG treated mice. (*n*=10 per group). Data from two independent experiments were pooled and presented. (F) Comparisons of the percentage of granulocyte monocyte progenitor cell(GMP) in Sca1- c-Kit+ Lineage- cells of PBS or BCG treated mice. (*n*=10 per group).Data from two independent experiments were pooled and presented. (G-L) Comparisons of the fold change of *Wdr5* (G), *Wdr5b* (H), *Kdm5a* (I), *Kdm5b* (J), *Kdm5c* (K) and *Kdm5d* (L) in RNA-sequencing data of PBS or BCG trained BMDM. (*n*=3 ). Data are presented as mean ± SEM. ns: non-significant, * *p* < 0.05, ** *p* < 0.01, *** *p* < 0.001 by the Student’s t test (B-L). Supplementary Fig S10 related to Figure 7. Upregulation of NOX2 complex in bladder with BCG-trained macrophage features after BCG treatment. (A-B) tSNE(A) and UMAP(B) dimensionality reduction analysis of macrophages in the bladder of BCG-treated group and control group. (C) Proportion of TAM1 and TAM2/3 macrophages in the bladder of BCG-treated group verse control group. (D) Volcano plots indicating the alterations of gene expressing profiles in the cells collected from the bladder in TAM1 verse TAM2/3. (E) Heatmaps showing relative expression of the genes involved in ROS generation by NOX2 complex and trained immunity signaling in TAM1 verse TAM2/3. (F-G) Comparisons of the fold change of NOX2 complex related genes (F) and trained immunity related genes (G) in RNA-sequencing data of NMIBC patients bladder. Data are presented as mean ± SEM. ns: non-significant, * *p* < 0.05, ** *p* < 0.01 by the One-way ANOVA (F-G). Supplementary Table S1. Clinical manifestations of NMIBC patients receiving BCG intravesical instillation with different responses. Supplementary Table S2. Key resources table.

## Data Availability

All data needed to evaluate the conclusions in the paper are present in the main text and/or the Supplementary information. Further data supporting the findings of this study is available upon reasonable request. Please contact the corresponding author.

## Abbreviations

ATAC-seq: Assay for Transposase-Accessible Chromatin sequencing
BCG: *Bacillus Calmette-Guerin*
BM: Bone Marrow
BMDM: Bone Marrow Derived Macrophage
DMSO: Dimethyl Sulfoxide
ELISA: Enzyme-Linked Immunosorbent Assay
GMP: Granulocyte Monocyte Progenitor
GSEA: Gene Set Enrichment Analysis
H3K4me3: Trimethylation of Histone H3 at lysine 4
H3K27ac: acetylation of Histone H3 at lysine 27
IL-1β: Interleukin − 1β
IL-10: Interleukin − 10
IFN-γ: Interferon -γ
LDH: Lactate Dehydrogenase
LLC: Lewis Lung Carcinoma
LPS: Lipopolysaccharide
LSK cells: Lineage^−^ Sca1^+^ cKit^+^ cells
mAb: monoclonal Antibody
MPP: Multipotent Progenitor
Mtb: *Mycobacterium tuberculosis*
mtROS: mitochondrial Reactive Oxygen Species
NAC: N-Acetylcysteine
NADPH: Nicotinamide adenine dinucleotide phosphate (reduced form)
NK cells: Natural killer cells
NMIBC: Non-Muscle Invasive Bladder Cancer
NOX2: NADPH Oxidase 2
PBS: Phosphate Buffered Saline
RNA-seq: RNA sequencing
ROS: Reactive Oxygen Species
scRNA-seq: single-cell RNA sequencing
ST-HSC: Short-Term Hematopoietic Stem Cells
TAM: Tumor Associated Macrophage
TCA cycle: Tricarboxylic Acid cycle
TGF-β: Transforming Growth Factor -β
TNF-α: Tumor Necrosis Factor -α
TME: Tumor Micro-Environment
UDC: Urine-Derived cells
WT: Wild Type

## References

[1] Netea MG, Domínguez-Andrés J, Barreiro LB, Chavakis T, Divangahi M, Fuchs E, et al. Defining trained immunity and its role in health and disease. Nat Rev Immunol. 2020;20:375–88. 32132681 10.1038/s41577-020-0285-6 PMC7186935

[2] Kleinnijenhuis J, Quintin J, Preijers F, Joosten LAB, Ifrim DC, Saeed S et al. Bacille Calmette-Guérin induces NOD2-dependent nonspecific protection from reinfection via epigenetic reprogramming of monocytes. Proc Natl Acad Sci. 2012;109:17537–17542. 10.1073/pnas.1202870109 PMC3491454 22988082

[3] Redelman-Sidi G, Glickman MS, Bochner BH. The mechanism of action of BCG therapy for bladder cancer—a current perspective. Nat Reviews Urol. 2014;11:153–62. 10.1038/nrurol.2014.15 24492433

[4] Bast Jr RC, Borsos BZT, Rapp HJ. BCG and cancer. N Engl. J Med. 1974;290:1458–69. 10.1056/NEJM197406272902605 4598691

[5] Hinshaw DC, Shevde LA. The Tumor Microenvironment Innately Modulates Cancer Progression. Cancer Res. 2019;79:4557–66. 31350295 10.1158/0008-5472.CAN-18-3962 PMC6744958

[6] Christofides A, Strauss L, Yeo A, Cao C, Charest A, Boussiotis VA. The complex role of tumor-infiltrating macrophages. Nat Immunol. 2022;23:1148–56. 35879449 10.1038/s41590-022-01267-2 PMC10754321

[7] Li Yang JH, Ren X, Carbone DP 1, Matrisian LM, Richmond A. Charles Lin, and Harold L. Moses. Abrogation of TGF beta signaling in mammary carcinomas recruits Gr-1 + CD11b+ myeloid cells that promote metastasis. Cancer Cell. 2008;13:23–35. 10.1016/j.ccr.2007.12.004 PMC2245859 18167337

[8] Wang R, Lu M, Chen H, Chen S, Luo X, Qin Y et al. Increased IL-10 mRNA expression in tumor-associated macrophage correlated with late stage of lung cancer. J Experimental Clin Cancer Res. 2011;30(1):62. 10.1186/1756-9966-30-62 PMC3117740 21595995

[9] Biswas SK, Mantovani A. Macrophage plasticity and interaction with lymphocyte subsets: cancer as a paradigm. Nat Immunol. 2010;11:889–96. 20856220 10.1038/ni.1937

[10] Lin Y, Xu J, Lan H. Tumor-associated macrophages in tumor metastasis: biological roles and clinical therapeutic applications. J Hematol Oncol. 2019;12(1):76. 10.1186/s13045-019-0760-3 PMC6626377 31300030

[11] Krausgruber T, Blazek K, Smallie T, Alzabin S, Lockstone H, Sahgal N, et al. IRF5 promotes inflammatory macrophage polarization and TH1-TH17 responses. Nat Immunol. 2011;12:231–8. 21240265 10.1038/ni.1990

[12] Nasir I, McGuinness C, Poh AR, Ernst M, Darcy PK. Britt. Tumor macrophage functional heterogeneity can inform the development of novel cancer therapies. Trends Immunol. 2023;44:971–85. 37995659 10.1016/j.it.2023.10.007

[13] Wu K, Lin K, Li X, Yuan X, Xu P, Ni P et al. Redefining Tumor-Associated Macrophage Subpopulations and Functions in the Tumor Microenvironment. Front Immunol. 2020;11:1731. 10.3389/fimmu.2020.01731 PMC7417513 32849616

[14] Wang J, Zhu N, Su X, Gao Y, Yang R. Novel tumor-associated macrophage populations and subpopulations by single cell RNA sequencing. Front Immunol. 2024;14:1264774. 10.3389/fimmu.2023.1264774 PMC10859433 38347955

[15] Garett Dunsmore WG, Li Z, Bejarano DA, Pai R, Yang K, Kwok I, Tan L, Ng M, Carlos, De La Fabregat C, Yatim A, Bougouin A, Mulder K, Villar JTJ, Bied M, Kloeckner BCharles-Antoine DutertreGrégoire GessainSvetoslav ChakarovZhaoyuan Liu, Scoazec J-Y. Ana-Maria Lennon-Dumenil, Thomas Marichal, Catherine Sautès-Fridman, Wolf Herman Fridman, Ankur Sharma, Bing Su, Andreas Schlitzer, Lai Guan Ng, Camille Blériot, Florent Ginhoux Timing and location dictate monocyte fate and their transition to tumor-associated macrophages. Sci Immunol. 2024;9:eadk3981. 10.1126/sciimmunol.adk3981 39058763

[16] DeNardo DG, Ruffell B. Macrophages as regulators of tumour immunity and immunotherapy. Nat Rev Immunol. 2019;19:369–82. 30718830 10.1038/s41577-019-0127-6 PMC7339861

[17] Fanucchi S, Domínguez-Andrés J, Joosten LAB, Netea MG. M M Mhlanga Intersection Epigenetics Metabolism Trained Immun Immun. 2021;54:32–43. 10.1016/j.immuni.2020.10.011 33220235

[18] Arts RJW, Carvalho A, La Rocca C, Palma C, Rodrigues F, Silvestre R, et al. Immunometabolic Pathways in BCG-Induced Trained Immunity. Cell Rep. 2016;17:2562–71. 27926861 10.1016/j.celrep.2016.11.011 PMC5177620

[19] Drummer C, Saaoud F, Shao Y, Sun Y, Xu K, Lu Y, et al. Trained Immunity and Reactivity of Macrophages and Endothelial Cells. Arterioscler Thromb Vasc Biol. 2021;41:1032–46. 33380171 10.1161/ATVBAHA.120.315452 PMC7904591

[20] Liemburg-Apers DC, Willems PHGM, Koopman WJH. and S. Grefte. Interactions between mitochondrial reactive oxygen species and cellular glucose metabolism. Arch Toxicol. 2015;89:1209–26. 10.1007/s00204-015-1520-y PMC4508370 26047665

[21] Rendra E, Riabov V, Mossel DM, Sevastyanova T, Harmsen MC. Kzhyshkowska. Reactive oxygen species (ROS) in macrophage activation and function in diabetes. Immunobiology. 2019;224:242–53. 30739804 10.1016/j.imbio.2018.11.010

[22] Herb M, Schramm M. Functions of ROS in Macrophages and Antimicrobial Immunity. Antioxidants. 2021;10(2):313. 10.3390/antiox10020313 PMC7923022 33669824

[23] Wang Y, Qi H, Liu Y, Duan C, Liu X, Xia T et al. The double-edged roles of ROS in cancer prevention and therapy. Theranostics. 2021;11:4839–57. 10.7150/thno.56747 PMC7978298 33754031

[24] Cheung EC, Vousden KH. The role of ROS in tumour development and progression. Nat Rev Cancer. 2022;22:280–97. 35102280 10.1038/s41568-021-00435-0

[25] Kotsafti A, Scarpa M, Castagliuolo I, Scarpa M. Reactive Oxygen Species Antitumor Immunity—From Surveillance Evasion Cancers. 2020;12(7):1748. 10.3390/cancers12071748 PMC7409327 32630174

[26] Ding C, Shrestha R, Zhu X, Geller AE, Wu S, Woeste MR, et al. Inducing trained immunity in pro-metastatic macrophages to control tumor metastasis. Nat Immunol. 2023;24:239–54. 36604547 10.1038/s41590-022-01388-8 PMC10636755

[27] Kalafati L, Kourtzelis I, Schulte-Schrepping J, Li X, Hatzioannou A, Grinenko T, et al. Innate Immune Training of Granulopoiesis Promotes Anti-tumor Activity. Cell. 2020;183:771–e78512. 33125892 10.1016/j.cell.2020.09.058 PMC7599076

[28] Arts RJW, S.J.C.F.M. Moorlag B, Novakovic Y, Li S-Y, Wang M, Oosting, et al. BCG Vaccination Protects against Experimental Viral Infection in Humans through the Induction of Cytokines Associated with Trained Immunity. Cell Host Microbe. 2018;23:89–e1005. 29324233 10.1016/j.chom.2017.12.010

[29] Zhang B-Z, Shuai H, Gong H-R, Hu J-C, Yan B, Yuen TT-T et al. Bacillus Calmette-Guérin–induced trained immunity protects against SARS-CoV-2 challenge in K18-hACE2 mice. JCI Insight. 2022;7(11):e157393. 10.1172/jci.insight.157393 PMC9220951 35446790

[30] Kaufmann E, Sanz J, Dunn JL, Khan N, Mendonça LE, Pacis A, et al. BCG Educates Hematopoietic Stem Cells to Generate Protective Innate Immunity against Tuberculosis. Cell. 2018;172:176–e19019. 29328912 10.1016/j.cell.2017.12.031

[31] Chu X, Tian Y, Lv C. Decoding the spatiotemporal heterogeneity of tumor-associated macrophages. Mol Cancer. 2024;23(1):150. 10.1186/s12943-024-02064-1 PMC11282869 39068459

[32] Liu Z, Gu Y, Chakarov S, Bleriot C, Kwok I, Chen X, et al. Fate Mapping via Ms4a3-Expression History Traces Monocyte-Derived Cells. Cell. 2019;178:1509–e152519. 31491389 10.1016/j.cell.2019.08.009

[33] Pedre B, Barayeu U, Ezeriņa D, Dick TP. The mechanism of action of N-acetylcysteine (NAC): The emerging role of H2S and sulfane sulfur species. Volume 228. Pharmacology & Therapeutics; 2021. 10.1016/j.pharmthera.2021.107916 34171332

[34] Moloney JN, Cotter TG. ROS signalling in the biology of cancer. Semin Cell Dev Biol. 2018;80:50–64. 28587975 10.1016/j.semcdb.2017.05.023

[35] Wang Y, Liu X-y, Wang Y, Zhao W-x, Li F-d, Guo P-r et al. NOX2 inhibition stabilizes vulnerable plaques by enhancing macrophage efferocytosis via MertK/PI3K/AKT pathway. Redox Biology. 2023;64:102763. 10.1016/j.redox.2023.102763 PMC10320254 37354827

[36] Shetty S, Kumar R, Bharati S. Mito-TEMPO, a mitochondria-targeted antioxidant, prevents N-nitrosodiethylamine-induced hepatocarcinogenesis in mice. Free Radical Biology Med. 2019;136:76–86. 10.1016/j.freeradbiomed.2019.03.037 30946961

[37] Noreng S, Ota N, Sun Y, Ho H, Johnson M, Arthur CP et al. Structure of the core human NADPH oxidase NOX2. Nat Commun. 2022;13(1):6079. 10.1038/s41467-022-33711-0 PMC9568551 36241643

[38] Cirovic B, de Bree LCJ, Groh L, Blok BA, Chan J, van der Velden WJFM, et al. BCG Vaccination in Humans Elicits Trained Immunity via the Hematopoietic Progenitor Compartment. Cell Host Microbe. 2020;28:322–e3345. 32544459 10.1016/j.chom.2020.05.014 PMC7295478

[39] Shilatifard A. Molecular implementation and physiological roles for histone H3 lysine 4 (H3K4) methylation. Curr. Opin Cell Biol. 2008;20:341–8. 10.1016/j.ceb.2008.03.019 PMC2504688 18508253

[40] Nhien Tran AB. Kai Ge. Lysine Demethylase KDM6A in Differentiation, Development, and Cancer. Mol Cell Biol. 2020;40:e00341–20. 32817139 10.1128/MCB.00341-20 PMC7523656

[41] Zhang C, Guan Y, Zou J, Yang X, Bayliss G. and S. Zhuang. Histone methyltransferase MLL1 drives renal tubular cell apoptosis by p53-dependent repression of E-cadherin during cisplatin-induced acute kidney injury. Cell Death Dis. 2022;13(9):770. 10.1038/s41419-022-05104-0 PMC9448773 36068197

[42] Wang X, Zhu K, Li S, Liao Y, Du R, Zhang X et al. MLL1, a H3K4 Methyltransferase, regulates the TNFα-stimulated activation of genes downstream of NF-κB. J Cell Sci. 2012;125(Pt17):4058–66. 10.1242/jcs.103531 22623725

[43] Wang H, Helin K. Roles of H3K4 methylation in biology and disease. Trends Cell Biol. 2025;35:115–28. 38909006 10.1016/j.tcb.2024.06.001

[44] Gauss KA, Nelson-Overton LK, Siemsen DW, Gao Y, DeLeo FR. Quinn. Role of NF-κB in transcriptional regulation of the phagocyte NADPH oxidase by tumor necrosis factor-α. J Leukoc Biol. 2007;82:729–41. 17537988 10.1189/jlb.1206735

[45] Anrather J, Racchumi G, Iadecola C. NF-κB Regulates Phagocytic NADPH Oxidase by Inducing the Expression of gp91. J Biol Chem. 2006;281:5657–67. 16407283 10.1074/jbc.M506172200

[46] Marcos Luengo-Blanco CP, Bustamante J, Walmir Cutrim Aragão-Filho, Paulo Vitor Soeiro Pereira, Jussara Rehder, Carolyn Padden, Casanova J-L, Peter E, Newburger. Antonio Condino-Neto. Essential role of nuclear factor-kappaB for NADPH oxidase activity in normal and anhidrotic ectodermal dysplasia leukocytes. Blood. 2008;112:1453-60. 10.1182/blood-2007-07-099267 PMC2515116 18523147

[47] Ma J, Liu J, Wang Z, Gu X, Fan Y, Zhang W et al. NF-kappaB-dependent MicroRNA-425 upregulation promotes gastric cancer cell growth by targeting PTEN upon IL-1β induction. Mol Cancer. 2014;13:40. 10.1186/1476-4598-13-40 PMC3941686 24571667

[48] Lopez-Beltran A, Cookson MS, Guercio BJ. and L. Cheng. Advances in diagnosis and treatment of bladder cancer. Bmj. 2024;384:e076743. 10.1136/bmj-2023-076743 38346808

[49] Daman AW, Antonelli AC, Redelman-Sidi G, Paddock L, Khayat S, Ketavarapu M, et al. Microbial cancer immunotherapy reprograms hematopoiesis to enhance myeloid-driven anti-tumor immunity. Cancer Cell. 2025;43:1442–e145910. 40446799 10.1016/j.ccell.2025.05.002 PMC12377364

[50] Tran MA, Youssef D, Shroff S, Chowhan D, Beaumont KG, Sebra R et al. Urine scRNAseq reveals new insights into the bladder tumor immune microenvironment. J Exp Med. 2024;221(8):e20240045. 10.1084/jem.20240045 PMC11157455 38847806

[51] Mantovani A, Marchesi F, Di Mitri D, Garlanda C. Macrophage Divers cancer dissemination metastasis Cell Mol Immunol. 2024;21:1201–14. 10.1038/s41423-024-01216-z PMC11528009 39402303

[52] Hedrick CC, Malanchi I. Neutrophils in cancer: heterogeneous and multifaceted. Nat Rev Immunol. 2021;22:173–87. 34230649 10.1038/s41577-021-00571-6

[53] Wu S-Y, Fu T, Jiang Y-Z. and Z.-M. Shao Nat killer cells cancer biology therapy Mol Cancer. 2020;19(1):120. 10.1186/s12943-020-01238-x PMC7409673 32762681

[54] Locati M, Curtale G, Mantovani A. Diversity, Mechanisms, and Significance of Macrophage Plasticity. Annual Rev Pathology: Mech Disease. 2020;15:123–47. 10.1146/annurev-pathmechdis-012418-012718 PMC7176483 31530089

[55] Epelman S, Kory J, Lavine, Gwendalyn J. Randolph Origin Funct Tissue Macrophages Immun. 2014;41:21–35. 10.1016/j.immuni.2014.06.013 PMC4470379 25035951

[56] Quail DF, Joyce JA. Microenvironmental regulation of tumor progression and metastasis. Nat Med. 2013;19:1423–37. 24202395 10.1038/nm.3394 PMC3954707

[57] Zhu Y, Herndon JM, Sojka DK, Kim K-W, Knolhoff BL, Zuo C et al. Tissue-Resident Macrophages in Pancreatic Ductal Adenocarcinoma Originate from Embryonic Hematopoiesis and Promote Tumor Progression. Immunity. 2017;47(2):323–38. 10.1016/j.immuni.2017.07.014 PMC5578409 28813661

[58] Ma R-Y, Black A. Qian. Macrophage diversity in cancer revisited in the era of single-cell omics. Trends Immunol. 2022;43:546–63. 35690521 10.1016/j.it.2022.04.008

[59] Aegerter H, Lambrecht BN, Jakubzick CV. Biology lung macrophages health disease Immun. 2022;55:1564–80. 10.1016/j.immuni.2022.08.010 PMC9533769 36103853

[60] Liu M, Ren Y, Zhou Z, Yang J, Shi X, Cai Y, et al. The crosstalk between macrophages and cancer cells potentiates pancreatic cancer cachexia. Cancer Cell. 2024;42:885–e9034. 38608702 10.1016/j.ccell.2024.03.009 PMC11162958

[61] Korbecki J, Kojder K, Simińska D, Bohatyrewicz R, Gutowska I, Chlubek D et al. CC Chemokines in a Tumor: A Review of Pro-Cancer and Anti-Cancer Properties of the Ligands of Receptors CCR1, CCR2, CCR3, and CCR4. International. J Mol Sci. 2020;21(21):8412. 10.3390/ijms21218412 PMC7665155 33182504

[62] Qian B-Z, Li J, Zhang H, Kitamura T, Zhang J, Campion LR, et al. CCL2 recruits inflammatory monocytes to facilitate breast-tumour metastasis. Nature. 2011;475:222–5. 21654748 10.1038/nature10138 PMC3208506

[63] Zhang J, Dong Y, Yu S, Hu K, Zhang L, Xiong M et al. IL-4/IL-4R axis signaling drives resistance to immunotherapy by inducing the upregulation of Fcγ receptor IIB in M2 macrophages. Cell Death Dis. 2024;15(7):500. 10.1038/s41419-024-06875-4 PMC11246528 39003253

[64] Lai Y, Yang X, Wei D, Wang X, Sun R, Li Y et al. BCG-trained macrophages couple LDLR upregulation to type I IFN responses and antiviral immunity. Cell Rep. 2025;44(4):115493. 10.1016/j.celrep.2025.115493 40178982

[65] Xu J-C, Huang Z-YCX-J, Wu J, Huang H, Niu L-F, Wang H-L, Li J-H, Douglas B, Lowrie Z, Lu HS-H. Multi-omics analysis reveals that linoleic acid metabolism is associated with variations of trained immunity induced by distinct BCG strains. Sci Adv. 2024;10:eadk8093. 38578989 10.1126/sciadv.adk8093 PMC10997199

[66] Quintin J, Saeed S, Joost HA, Martens, Evangelos J, Giamarellos-Bourboulis DC, Ifrim C, Logie, et al. Candida albicans Infection Affords Protection against Reinfection via Functional Reprogramming of Monocytes. Cell Host Microbe. 2012;12:223–32. 22901542 10.1016/j.chom.2012.06.006 PMC3864037

[67] Lange C, Aaby P, Behr MA, Donald PR, Kaufmann SHE, Netea MG, et al. 100 years of Mycobacterium bovis bacille Calmette-Guérin. Lancet Infect Dis. 2022;22:e2–12. 34506734 10.1016/S1473-3099(21)00403-5 PMC11967564

[68] Singh AK, Netea MG, Bishai WR. BCG turns 100: its nontraditional uses against viruses, cancer, and immunologic diseases. J Clin Invest. 2021;131(11):e148291. 10.1172/JCI148291 PMC8159679 34060492

