# Supplementary material for: BCG-trained macrophages promote pan-anti-tumor activity through epigenetic rewiring of NOX2-ROS axis
Source: J Exp Clin Cancer Res. 2026 Apr 24;45:134. doi: 10.1186/s13046-026-03708-4 (PMC13261971; doi:10.1186/s13046-026-03708-4)
Supplement: Supplementary file 1 — Supplementary Material 1. Supplementary Fig S1 related to Figure 1. BCG-trained mice exhibits enhanced TNF-α and IL-1β production and long period of anti-tumor effects. (A) Experimental scheme of TNF-α and pro-IL1β production of macrophage in spleen in PBS or BCG treated mice with or without LPS re-stimulation. (B-C) Comparison of TNF-α+(B) and pro-IL1β+(C) macrophage in spleen in PBS or BCG treated mice with or without LPS re-stimulation(n=5 per group). Data from two independent experiments were pooled and presented. (D) Experimental scheme. Briefly, BCG was intraperitoneally injected once and multiple subcutaneous grafted tumors were established after 6 weeks. Tumor growth curves were monitored and mice were sacrificed according to tumor volumes. (E-F) Tumor growth curves of grafted Lewis lung carcinoma (LLC) (E) and tumor weight (F) in PBS (n = 4) versus BCG treated (n =4) mice. (G) CD8+ T cells gating strategy. (H) Representative contour map and comparisons of the percentages of CD8+ T cells in CD45+ cells in LLC grafted tumors between PBS and BCG-treated mice with or without anti-F4/80 mAb treatment. (I-L) Representative contour map and comparisons of IFN-γ+(I), TNF-α+(J), CD107A+(K) and Granzyme B+(L) CD8+ T cells in LLC grafted tumors between PBS and BCG-treated mice with or without anti-F4/80 mAb treatment. Data are presented as mean ± SEM. ns: non-significant, * p < 0.05, ** p < 0.01, *** p < 0.001, **** p < 0.0001 by the Student’s t test (E-F) and one-way ANOVA (B,C,H-L). Supplementary Fig S2 related to Fig. 1. BCG-trained mice exhibit enhanced anti-tumor effects depending on BCG-trained macrophages. (A-D) Experimental scheme of anti-tumor immunity in PBS or BCG-trained WT or nude mice. Briefly, PBS or BCG was injected once and mouse colorectal cell line MC38 was inoculated in C57BL/6 and nude mice (A). Tumor growth curves (B) were monitored every 2 days. Tumor weight (C) and the percentage of CD8+T cells (D) were assayed at Day 16 after the sacrifice of P [file 13046_2026_3708_MOESM1_ESM.docx]

**Supplementary Data**

**Supplementary Figure 1 related to Figure 1. BCG-trained mice exhibits enhanced TNF-α and IL-1β production and long period of anti-tumor effects.**

(A) Experimental scheme of TNF-α and pro-IL1β production of macrophage in spleen in PBS or BCG treated mice with or without LPS re-stimulation.

(B-C) Comparison of TNF-α^+^(B) and pro-IL1β^+^(C) macrophage in spleen in PBS or BCG treated mice with or without LPS re-stimulation(n=5 per group). Data from two independent experiments were pooled and presented.

(D) Experimental scheme. Briefly, BCG was intraperitoneally injected once and multiple subcutaneous grafted tumors were established after 6 weeks. Tumor growth curves were monitored and mice were sacrificed according to tumor volumes.

(E-F) Tumor growth curves of grafted Lewis lung carcinoma (LLC) (E) and tumor weight (F) in PBS (n = 4) versus BCG treated (n =4) mice.

(G) CD8^+^ T cells gating strategy.

(H) Representative contour map and comparisons of the percentages of CD8^+^ T cells in CD45^+^ cells in LLC grafted tumors between PBS and BCG-treated mice with or without anti-F4/80 mAb treatment.

(I-L) Representative contour map and comparisons of IFN-γ^+^(I), TNF-α^+^(J), CD107A^+^(K) and Granzyme B^+^(L) CD8^+^ T cells in LLC grafted tumors between PBS and BCG-treated mice with or without anti-F4/80 mAb treatment.

Data are presented as mean ± SEM. ns: non-significant, * p < 0.05, ** p < 0.01, *** p < 0.001, **** p < 0.0001 by the *Student’s t* test (E-F) and one-way ANOVA (B,C,H-L).

**Supplementary Figure 2 related to Figure 1. BCG-trained mice exhibit enhanced anti-tumor effects depending on BCG-trained macrophages.**

(A-D) Experimental scheme of anti-tumor immunity in PBS or BCG-trained WT or nude mice. Briefly, PBS or BCG was injected once and mouse colorectal cell line MC38 was inoculated in C57BL/6 and nude mice (A). Tumor growth curves (B) were monitored every 2 days. Tumor weight (C) and the percentage of CD8^+^T cells(D) were assayed at Day 16 after the sacrifice of PBS and BCG-trained C57BL/6 and nude mice(n=4 per group).

(E-H) Experimental scheme of anti-tumor immunity in BCG-trained mice with the depletion of CD8^+^T cells. Briefly, BCG was injected once and mouse LLC cell lines was inoculated in C57BL/6 mice at Day 7. Anti-mouse CD8 antibodies were injected one day before LLC inoculation (Day -1) and Day 6, 13 (E). Tumor growth curves(F) were monitored every 2 days.Tumor weight (G) and the percentage of CD8^+^T cells(H) were assayed at Day 18 after the sacrifice of PBS and BCG-trained mice with or without anti-CD8 mAb treatment(n=3 per group).

(I-L) Experimental scheme of anti-tumor immunity in BCG-trained mice with the depletion of NK cells. Briefly, BCG was injected once and mouse LLC cell lines was inoculated in C57BL/6 mice at Day 7. Anti-mouse NK1.1 antibodies were injected one day before LLC inoculation (Day -1) and Day 4, 9, 12 (I). Tumor growth curves(J) were monitored every 2 days.Tumor weight (K) and the percentage of NK cells(L) were assayed at Day 18 after the sacrifice of PBS and BCG-trained mice with or without anti-NK1.1 mAb treatment(n=3 per group).

(M-P) Experimental scheme of anti-tumor immunity in BCG-trained mice with the depletion of neutrophils. Briefly, BCG was injected once and mouse LLC cell lines was inoculated in C57BL/6 mice at Day 7. Anti-mouse Ly6G antibodies were injected one day before LLC inoculation (Day -1) and Day 2, 5, 8, 10 and 15 (M). Tumor growth curves(N) were monitored every 2 days.Tumor weight (O) and the percentage of neutrophils(P) were assayed at Day 18 after the sacrifice of PBS and BCG-trained mice with or without anti-Ly6G mAb treatment(n=4 per group).

Data are presented as mean ± SEM. ns: non-significant, * p < 0.05, ** p < 0.01, *** p < 0.001, **** p < 0.0001 by one-way ANOVA (B-P).

**Supplementary Figure 3 related to Figure 1. BCG-trained macrophages exhibits enhanced TNF-α and IL-1β production after LPS re-stimulation and higher cytotoxicity to LLC and MC38 cells.**

(A) Experimental scheme of TNF-α and pro-IL1β production of bone marrow derived macropage(BMDM) control or BCG-trained BMDM with or without LPS re-stimulation.

(B-C) Comparison of TNF-α(B) and IL1β(C) production in BMDM control or BCG-trained BMDM with or without LPS re-stimulation. Data were pooled from 3 individual mice.

(D) Experimental scheme of *in vitro* cytotoxicity of BCG-treated bone marrow derived macrophages (BMDMs) against tumor cells. Briefly, BMDMs were induced by M-CSF for 6 days and treated with BCG lysates for 24 hrs. BMDMs were washed once with PBS and rested for 3 days. Then BMDMs were incubated with tumor cell lines at 5:1 ratios of effector to target cells. The culture supernatants were collected 4 hrs later and subjected to determine the LDH level according to the manufacturer’s instructions.

(E) Comparison of the cytotoxicity of BMDMs to LLC and MC38 tumor cells with or without BCG treatment. Data were pooled from 4 individual mice and presented

(F) Experimental scheme of TNF-α and pro-IL1β production of control or BCG-trained Raw264.7 cells with or without LPS re-stimulation.

(G-H) Comparison of TNF-α(G) and IL1β(H) production in control or BCG-trained Raw264.7 with or without LPS re-stimulation.

(I) Experimental scheme of the effects on *in vitro* cytotoxicity of control or BCG-treated RAW264.7 cells using LDH release assay.

(J) Comparisons of *in vitro* cytotoxicity of control or BCG-trained RAW264.7 cells to LLC cells or MC38 cells.

Data are presented as mean ± SEM. ns: non-significant, * p < 0.05, ** p < 0.01, *** p < 0.001, **** p < 0.0001 by the *Student’s t* test (E and J) and one-way ANOVA(B,C,G and H).

**Supplementary Figure 4 related to Figure 2. BCG-trained mo-macrophages exhibits more activated characteristics.**

(A) Gating strategy.

(B-H) Representative flow contour maps and comparisons of CD80^+^(B), CD80^+^(C), MHC-II^+^(D), CD163^+^(E), CD206^+^(F), pro-IL1β^+^(G) and TNF-α^+^(H) mono-macrophages in LLC grafted tumors between PBS and BCG-treated mice.

Data are presented as mean ± SEM. * p < 0.05, ** p < 0.01, *** p < 0.001 by the *Student’s t* test (B-H).

**Supplementary Figure 5 related to Figure 2. BCG induced increased production of TNF-α and pro-IL1β macrophages more produced by TAM1 and bone marrow derived macrophages.**

(A-B) Representative flow contour maps and comparisons of the percentages of pro-IL1β^+^(A) and TNF-α^+^(B)TAMs in TAM subsets in LLC grafted tumors between PBS(n=3) and BCG-treated mice(n=3).

(C-D) Representative flow contour maps and comparisons of the percentages of pro-IL1β^+^(C) and TNF-α^+^(D)TAMs in Ms4a3^+^ or Ms4a3**^-^** macrophages in LLC grafted tumors between PBS(n=3) and BCG-treated mice(n=3).

(E) Experiment scheme.

(F-I) Comparisons of the percentages of CCR2^+^(F), CD64^+^(G), CD11c^+^(H) and CD73^+^(I) monocytes in bone marrow between PBS(n=3) and BCG-treated mice(n=3).

Data are presented as mean ± SEM.ns: non-significant,* p < 0.05,** p < 0.01, *** p < 0.001 by the *Student’s t* test (A-I).

**Supplementary Figure 6 related to Figure 2. CCR2 drives BCG trained macrophages tumor infiltration and TAM1 generation.**

(A) Experimental scheme of BMDM transfer capacity assay by transwell system. Briefly, BMDMs were trained with PBS or BCG lysate for 24 hours. After wash the supernatant and rest for 2 days, 50000 BMDMs were inoculated on the upper chamber and 25000 LLC cells were inoculated on the lower chamber. The cells were treated with DMSO or 1μM CCR2 inhibitor PF-4136309 for 48 hours. The cells in lower chamber were digested and flow cytometry were used to determine the number of transferred BMDMs.

(B) Comparison of the number of transferred BMDMs in lower chamber in the transwell system. Data were pooled from 3 individual mice.

(C) Experimental scheme of *in vivo* anti-tumor effects in PBS or BCG-trained mice with or without CCR2 inhibitor PF-4136309.

(D-E) Determination of tumor growth curves (D) and tumor weight (E) of LLC grafted tumors in PBS and BCG-trained mice with or without the injection of PF-4136309 (n=3 per group).

(F-J) Percentages of Monocytes(F), TAMs(G), TAM1(H), TAM2(I) and TAM3(J) in CD45^+^ cells in LLC grafted tumors from PBS and BCG-trained mice with or without the injection of PF-4136309 (n=3 per group).

Data are presented as mean ± SEM. ns: non-significant, * p < 0.05, ** p < 0.01, *** p < 0.001, **** p < 0.0001 by the One-way ANOVA(B-J).

**Supplementary Figure 7 related to Figure 3. BCG-trained macrophages exhibit enhanced anti-tumor cytotoxicity relying on ROS production.**

(A,C) Experimental scheme of ROS detection between control and BCG-trained BMDM(A) or Raw264.7 cells(C) upon LPS re-stimulation.

(B,D) Comparison of the percentage of ROS-producing BMDMs(B) or Raw264.7 cells(D) with or without BCG treatment upon LPS re-stimulation.

(E) Experimental scheme of the effects on *in vitro* cytotoxicity of control or BCG-treated RAW264.7 cells using LDH release assay and ROS detection.

(F) Comparisons of *in vitro* cytotoxicity of control or BCG-trained RAW264.7 cells to LLC cells with or without NAC treatment.

(G) Comparisons of the percentages of ROS production in control or BCG-trained RAW264.7 cells with or without NAC treatment.

(H) Comparisons of the percentages of macrophages in CD45^+^ cells in LLC grafted tumors between PBS and BCG-treated mice with or without NAC treatment(n=4 per group).

(I-L) Comparisons of CD80^+^(I), CD86^+^(J), pro-IL1β^+^(K) and TNF-α^+^(L) macrophages in LLC grafted tumors between PBS and BCG-treated mice with or without NAC treatment.

(M) Comparisons of the percentages of CD8^+^ T cells in CD45^+^ cells in LLC grafted tumors between PBS and BCG-treated mice with or without NAC treatment(n=4 per group).

(N-Q) Comparisons of IFN-γ^+^(N), TNF-α^+^(O), CD107A^+^(P) and Granzyme B^+^(Q) CD8^+^ T cells in LLC grafted tumors between PBS and BCG-treated mice with or without NAC treatment(n=4 per group).

Data are presented as mean ± SEM. ns: non-significant, * p < 0.05, ** p < 0.01, *** p < 0.001, **** p < 0.0001 by the One-way ANOVA (B-Q).

**Supplementary Figure 8 related to Figure 4. BCG-trained BMDMs maintain normal mitochondrial structure and BCG-trained RAW264.7 generate extra ROS in a NOX2 complex-dependent manner.**

(A) Mitochondrial structures of BMDMs with or without BCG treatment by transmission electron microscopy.

(B-C) Comparison of mitochondrial length (B) and numbers (C) between control and BCG-trained BMDMs.

(D) Experimental scheme of *in vitro* cytotoxicity assay in BCG-trained RAW264.7 that was affected by NOX2 oxidase activity by the LDH release assay.

(E) Comparisons of *in vitro* cytotoxicity of PBS and BCG-trained RAW264.7 to LLC cells with or without GSK2795039 treating.

(F) Comparisons of the percentages of ROS-producing RAW264.7 cells with PBS or BCG treatment at the presence of vehicle and GSK2795039.

(G) Experimental scheme of *in vitro* cytotoxicity assay in BCG-trained Raw264.7 that was affected by NOX2 complex gene expression through the LDH release assay.

(H) Comparisons of *in vitro* cytotoxicity of PBS and BCG-trained Raw264.7 against LLC cells with or without the interference of *Cyba* and *Cybb* expressions (n = 3).

(I) Comparisons of the percentages of ROS-producing Raw264.7 with or without the interference of *Cyba* and *Cybb* expressions (n = 3).

(J) Experimental scheme of co-transferring of LLC tumor cells and PBS/BCG-trained BMDMs with mock or *Cybb* siRNA transfection in C57BL/6 mice(n = 4 per group).

(K-L) Comparisons of tumor growth curves (K) and tumor weight (L) of mice inoculated by LLC cells mixed with PBS/BCG-trained BMDM with mock or *Cybb* siRNA transfection (n = 4 per group).

Data are presented as mean ± SEM. ns: non-significant, * p < 0.05, ** p < 0.01, *** p < 0.001, **** p < 0.0001 by the *Student’s t* test(B-C) and One-way ANOVA(E-L).

**Supplementary Figure 9 related to Figure 5. Increased proportion of bone marrow hematopoietic stem cells in mice treated with BCG.**

(A-B) Representative flow contour maps(A) and quantifications (B) of the percentages of Lineage^-^Sca-1^+^c-Kit^+^(LSK) cells in lineage^-^bone marrow cells of PBS or BCG treated mice.(n=10 per group).Data from two independent experiments were pooled and presented.

(C-E) Comparisons of the percentage of LSK(C), multi-potent progenitor (MPP)(D), short-term hematopoietic stem cells(ST-HSC)(E) in total bone marrow cells of PBS or BCG treated mice. (n=10 per group).Data from two independent experiments were pooled and presented.

(F) Comparisons of the percentage of granulocyte monocyte progenitor cell(GMP) in Sca1- c-Kit+ Lineage- cells of PBS or BCG treated mice. (n=10 per group).Data from two independent experiments were pooled and presented.

(G-L) Comparisons of the fold change of *Wdr5*(G), *Wdr5b*(H), *Kdm5a*(I), *Kdm5b*(J), *Kdm5c*(K) and *Kdm5d*(L) in RNA-sequencing data of PBS or BCG trained BMDM. (n=3 ).

Data are presented as mean ± SEM. ns: non-significant, * p < 0.05, ** p < 0.01, *** p < 0.001 by the *Student’s t* test (B-L).

**Supplementary Figure 10 related to Figure 7. Upregulation of NOX2 complex in bladder with BCG-trained macrophage features after BCG treatment.**

(A-B) tSNE(A) and UMAP(B) dimensionality reduction analysis of macrophages in the bladder of BCG-treated group and control group.

(C) Proportion of TAM1 and TAM2/3 macrophages in the bladder of BCG-treated group verse control group.

(D) Volcano plots indicating the alterations of gene expressing profiles in the cells collected from the bladder in TAM1 verse TAM2/3.

(E) Heatmaps showing relative expression of the genes involved in ROS generation by NOX2 complex and trained immunity signaling in TAM1 verse TAM2/3.

(F-G) Comparisons of the fold change of NOX2 complex related genes(F) and trained immunity related genes(G) in RNA-sequencing data of NMIBC patients bladder.

Data are presented as mean ± SEM. ns: non-significant, * p < 0.05, ** p < 0.01 by the One-way ANOVA (F-G).

Supplementary Table1 Clinical manifestations of NMIBC patients receiving BCG intravesical instillation with different responses

| Characteristics | BCG responder (n=9) | BCG non-responder (n=4) | *p* value |
| --- | --- | --- | --- |
| Age | 66.22 ± 4.284 | 67.00 ± 5.986 | 0.920 |
| Gender(Male/Female) | 8/1 | 3/1 | 0.561 |
| CRP (mg/L) | 2.667 ± 0.897 | 3.000 ± 1.225 | 0.837 |
| WBC (10^9^/L) | 7.007 ± 0.785 | 5.475 ± 0.912 | 0.276 |
| RBC (10^12^/L) | 4.498 ± 0.183 | 4.238 ± 0.097 | 0.386 |
| HGB (g/L) | 139.6 ± 4.981 | 127.5 ± 1.708 | 0.147 |
| HCT (%) | 41.18 ± 1.409 | 37.90 ± 0.707 | 0.166 |
| PLT (10^9^/L) | 198.7 ± 48.31 | 179.0 ± 17.95 | 0.485 |
| NEUT# (10^9^/L) | 4.083 ± 0.527 | 3.525 ± 0.670 | 0.553 |
| MONO# (10^9^/L) | 0.531 ± 0.052 | 0.375 ± 0.075 | 0.120 |
| LYMPH# (10^9^/L) | 2.147 ± 0.318 | 1.450 ± 0.206 | 0.195 |

CRP: C-reactive protein, WBC: white blood cell, RBC: red blood cell, HGB: hemoglobin, HCT: haematocrit, PLT: platelet, NEUT# : absolute number of neutrophils, MONO# : absolute number of monocytes, LYMPH# : absolute number of lymphocytes.

Supplementary Table2 Key resources table

| **REAGENT or RESOURCE** | **SOURCE** | **IDENTIFIER** |
| --- | --- | --- |
| **Antibodies** | | |
| CD45 Monoclonal Antibody (30-F11), Alexa Fluor™ 700 | Thermo | Cat# 56-0451-82, RRID:AB_891454 |
| APC/Cyanine7 anti-mouse CD45 | BioLegend | Cat# 103116, RRID:AB_312981 |
| APC anti-mouse/human CD11b | BioLegend | Cat#101211, RRID:AB_312794 |
| BUV395 Rat Anti-CD11b | BD Biosciences | Cat# 565976, RRID:AB_2721166 |
| BV421 Rat Anti-Mouse F4/80 | BD Biosciences | Cat# 565411, RRID:AB_2734779 |
| Brilliant Violet 785 anti-mouse Ly-6G | BioLegend | Cat# 127645, RRID:AB_2566317 |
| BV605 Rat Anti-Mouse Ly-6C | BD Biosciences | Cat# 563011, RRID:AB_2737949 |

| FITC anti-mouse CD80 | BioLegend | Cat# 104705, RRID:AB_313126 |
| --- | --- | --- |
| Rat Anti-CD86 Monoclonal Antibody | BD Biosciences | Cat# 553692, RRID:AB_394994 |

| PerCP-Cy5.5 Rat Anti-Mouse I-A/I-E (M5/114.15.2) | BD Biosciences | Cat# 562363, RRID:AB_11153297 |
| --- | --- | --- |
| Alexa Fluor(R) 700 anti-mouse I-A/I-E | BioLegend | Cat# 107622, RRID:AB_493727 |
| Brilliant Violet 711 anti-mouse CD64 | BioLegend | Cat# 139311, RRID:AB_2563846 |
| APC anti-mouse CD192(CCR2) | BioLegend | Cat# 150627, RRID:AB_2810414 |
| PercP-Cyanine5.5 anti-mouse CD73 | BioLegend | Cat# 127214, RRID:AB_11219403 |
| Brilliant Violet 510 anti-mouse CD11c | BioLegend | Cat# 117353, RRID:AB_2686978 |
| Ms CD11c FITC HL3 | BD Biosciences | Cat# 557400, RRID:AB_396683 |

| Brilliant Violet 650 anti-mouse CD206 (MMR) | BioLegend | Cat# 141723, RRID:AB_2562445 |
| --- | --- | --- |
| [APC/Cyanine7 anti-mouse CD163](https://scicrunch.org/resources/data/record/nif-0000-07730-1/AB_2936556/resolver?q=*&l=*&filter%5b%5d=Catalog%20Number:155324&i=rrid:ab_2936556-2872012) | BioLegend | Cat# 155324, RRID:AB_2936556 |

| Streptavidin PercP-Cyanine5.5 conjugate | Thermo | Cat# 45-4317-82, RRID:AB_10311495 |
| --- | --- | --- |
| Ly-6A/E (Sca-1) Monoclonal Antibody (D7), FITC | Thermo | Cat# 11-5981-85, RRID:AB_465334 |
| CD117 (c-Kit) Monoclonal Antibody (ACK2), APC | Thermo | Cat# 17-1172-82, RRID:AB_469433 |
| Brilliant Violet 510 anti-mouse CD48 | BioLegend | Cat# 103443, RRID:AB_2650826 |
| PE/Cyanine7 anti-mouse CD150 (SLAM) | BioLegend | Cat# 115914, RRID:AB_439797 |
| CD34 Monoclonal Antibody (RAM34), eFluor™ 450 | Thermo | Cat# 48-0341-82, RRID:AB_2043837 |
| CD16/CD32 Monoclonal Antibody (93), PE | Thermo | Cat# 12-0161-82, RRID:AB_465568 |
| BV711 Rat Anti-Mouse TNF(MP6-XT22) | BD Biosciences | Cat# 563944, RRID:AB_2738499 |
| Brilliant Violet 650™ anti-mouse TNF-α | BioLegend | Cat# 506333, RRID:AB_2562450 |
| IL-1 beta (Pro-form) Monoclonal Antibody (NJTEN3), PE-Cyanine7 | Thermo | Cat# 25-7114-82 , RRID:AB_2573526 |
| BV711 Hamster Anti-Mouse CD3e (145-2C11) | BD Biosciences | Cat# 563123; RRID: AB_2687954 |
| CD8a Monoclonal Antibody (53-6.7), PE-Cyanine7 | Thermo | Cat# 25-0081-82, RRID:AB_469584 |
| Ms NK1.1 BV650 PK136 | BD Biosciences | Cat# 564143, RRID:AB_2738617 |
| Ms IFN-Gma APC-Cy7 XMG1.2 | BD Biosciences | Cat# 561479, RRID:AB_10898181 |
| APC anti-mouse TNF-α | BioLegend | Cat# 506308, RRID:AB_315429 |
| FITC Rat Anti-Mouse CD107a (1D4B) | BD Biosciences | Cat# 553793; RRID: AB_395057 |
| PE Rat Anti-Mouse Granzyme B (NGZB) | Thermo | Cat# 12-8898-82; RRID: AB_10870787 |
| Streptavidin PercP-Cyanine5.5 conjugate | Thermo | Cat# 45-4317-82, RRID:AB_10311495 |
| Ly-6A/E (Sca-1) Monoclonal Antibody （D7), FITC | Thermo | Cat# 11-5981-85, RRID:AB_465334 |
| CD117 (c-Kit) Monoclonal Antibody （ACK2), APC | Thermo | Cat# 17-1172-82, RRID:AB_469433 |
| Brilliant Violet 510(TM) anti-mouse CD48 | BioLegend | Cat# 103443, RRID:AB_2650826 |
| PE/Cyanine7 anti-mouse CD150 (SLAM) | BioLegend | Cat# 115914, RRID:AB_439797 |
| CD135 (Flt3) Monoclonal Antibody (A2F10), PE | Thermo | Cat# 12-1351-82, RRID:AB_465859 |
| CD34 Monoclonal Antibody (RAM34), eFluor™ 450 | Thermo | Cat# 48-0341-82, RRID:AB_2043837 |
| CD16/CD32 Monoclonal Antibody (93), PE | Thermo | Cat# 12-0161-82, RRID:AB_465568 |
| Recombinant Anti-CD68 antibody | Abcam | Cat# ab213363, RRID:AB_2801637 |
| Recombinant Anti-CD64 antibody | Abcam | Cat# ab302901 |
| CCR2 Polyclonal antibody | Thermo | Cat# PA5-23037, RRID:AB_11153363 |
| Recombinant Anti-NOX2 antibody | Abcam | Cat# ab311337 |
| NOX2 Antibody | Affinity Biosciences | Cat# DF6520, RRID:AB_2838482 |
| CYBA Antibody | Affinity Biosciences | Cat# DF10099, RRID:AB_2840679 |
| NOXA2/p67phxo Antibody | AiFang biological | Cat# AF301903 |
| Phospho-NCF2 (Thr233) Antibody | Affinity Biosciences | Cat# AF4343, RRID:AB_2844422 |
| Rac1 Antibody | Proteintech | Cat# 24072-1-AP, RRID:AB_2879427 |
| Anti-rabbit IgG,HRP-linked Antibody | CST | Cat# 7074S |
| Phospho-NCF2 (Thr233) Antibody | Affinity Biosciences | Cat# AF4343, RRID:AB_2844422 |
| NF-kappaB p65(D14E12)XP(R) Rabbit mAb | CST | Cat# 8242, RRID:AB_10859369 |
| Phospho-NF-κB p65 (Ser536) Rabbit Polyclonal Antibody | Beyotime Biotechnology | Cat# AF5881 |
| Rabbit Anti-Mouse β-Actin (13E5) | CST | Cat# 4970, RRID: AB_2223172 |
| Alexa Fluor® 647 Donkey anti-rabbit IgG (minimal x-reactivity) Antibody | BioLegend | Cat# 406414, RRID:AB_2563202 |
| InVivoMab anti-mouse CD8α | Bioxcell | Cat# BE0061, RRID:AB_1125541 |
| InVivoPlus anti-mouse NK1.1 | Bioxcell | Cat# BE0036, RRID:AB_1107737 |
| InVivoMAb anti-mouse F4/80 | Bioxcell | Cat# BE0206, RRID:AB_10949019 |
| InVivoPlus anti-mouse Ly6G | Bioxcell | Cat# BE0075, RRID:AB_1107721 |
| **Bacterial Strains** | | |
| Bacillus Calmette-Guérin (Japan strain) | Gifted from *Dr.* Xiaoyong Fan, Fudan University | N/A |
| **Chemicals, Reagents and Recombinant Proteins** | | |
| Fixation/Permeabilization Solution Kit | BD Biosciences | Cat# 554715 |
| Mouse Hematopoietic Progenitor Cell Enrichment Set-DM | BD Biosciences | Cat# 558451 |
| Mouse TNF alpha Uncoated ELISA | Thermo | Cat# 88-7324-88 |
| Mouse IL-1 beta Uncoated ELISA | Thermo | Cat# 88-7013-88 |
| Tumor Dissociation Kit | MiltenyiBiotec | Cat# 130-096-730 |
| Cytotoxicity LDH Assay Kit-WST | Dojindo | Cat# CK12 |
| ROS Assay Kit -Highly Sensitive DCFH-DA | Dojindo | Cat# R252 |
| MitoSOX Red superoxide indicators | Thermo | Cat# M36008 |

| Omni-Easy™ One-step Color PAGE Gel Rapid Preparation Kit | Epizyme | Cat# PG212 |
| --- | --- | --- |
| Enhanced BCA Protein Assay Kit | Beyotime Biotechnology | Cat# P0009 |
| Tris/Glycine/SDS Running Buffer(10×) | Epizyme | Cat# PS105 |
| Omni-Flash™ Western Blot Rapid Transfer Buffer(10×) | Epizyme | Cat# PS201 |
| Protein Free Rapid Blocking Buffer(1×) | Epizyme | Cat# PS108P |

| Universal Antibody Dilution Buffer | Epizyme | Cat# PS119L |
| --- | --- | --- |
| Rapid stripping buffer solution | Epizyme | Cat# PS107P |
| CALNP™ RNAi in vitro | D-Nano Therapeutics | Cat#: DN001 |
| PrimeScript™ RT reagent Kit | Takara | Cat# RR037B |
| TB Green® Premix Ex Taq™ II (Tli RNase H Plus) | Takara | Cat# RR820A |
| High-sig ECL Substrate (ECL) | Tanon | Cat# 180-5001 |
| Hyperactive pG-MNase  CUT&RUN Assay Kit for PCR/qPCR | Vazyme | Cat# HD101 |
| Hyperactive ATAC-Seq Library Prep Kit for Illumina | Vazyme | Cat# TD711 |
| EasySep™ Mouse Hematopoietic Progenitor Cell Isolation Kit | Stemcell | Cat# 19856 |
| Opti-MEM | Gibco | Cat# 31985070 |
| Accutase Cell Detachment Solution | Biolegend | Cat# 423201 |
| Phosphatase inhibitor Cocktail | TargetMol | Cat# C0003 |
| Protease Inhibitor Cocktail | TargetMol | Cat# C0001 |
| Mouse CSF-1/M-CSF Protein | Abclonal | Cat# RP01216- |
| Dulbecco’s Modified Eagles Medium | Gibco | Cat# 11965-092 |
| RPMI 1640 Medium | Gibco | Cat# 11875-093 |
| Fetal Bovine Serum (FBS) | Bio-Channel | Cat# BC-SE-FBS01 |
| L-glutamine | Gibco | Cat# 25030081 |
| MEM Non-Essential Amino Acids Solution (100X) | Gibco | Cat# 11140035 |
| 2-mercaptoethanol | Gibco | Cat# 21985023 |
| Penicillin-streptomycin (PS) | Gibco | Cat# 15070-063 |
| HEPES | Gibco | Cat# 15630080 |
| Phosphate Buffered Saline | HyClone | Cat# SH30256.01 |
| 0.05% Trypsin + 0.53 mM EDTA | Gibco | Cat# 25300-062 |
| LPS from E.coli O55:B5 | Sigma-Aldrich | Cat# L2880 |
| N-acetylcysteine | Sigma | Cat# A9165 |
| PF-4136309 | MCE | Cat# HY-13245 |
| GSK2795039 | Selleck | Cat# S8974 |
| Mito-tempo | Selleck | Cat# S9733 |
| TPCA-1 | Selleck | Cat# S2824 |
| MM102 | Selleck | Cat# S7265 |
| [20X TBS B](https://store.sangon.com/productDetail?productInfo.code=B548105)uffer | Sangon Biotech | Cat# [B548105](https://store.sangon.com/productDetail?productInfo.code=B548105) |
| Tween-80 | Sangon Biotech | Cat# A600562 |
| Bovine Serum Albumin (BSA) | Gibco | Cat# 15260-011 |
| RIPA Lysis and Extraction Buffer (RIPA) | Invitrogen | Cat# 89900 |
| Dimethyl Sulfoxide（DMSO） | Sigma-Aldrich | Cat# D2650 |
| Difco Middlebrook 7H9 Broth | BD Biosciences | Cat# 271310 |
| Middlebrook OADC Enrichment | BBL | Cat# 212351 |
| Glycerol | Sangon Biotech | Cat# A600232 |
| Tween-20 | Sangon Biotech | Cat# [A600560](https://store.sangon.com/productDetail?productInfo.code=A600560) |
| TRIzol | Thermo | Cat# 15596026 |
| **Cell Lines** | | |
| Mouse Lewis lung carcinoma cell line | Cell bank of the Chinese academy of sciences | N/A |
| Mouse B16-F10 melanoma cells | Cell bank of the Chinese academy of sciences | N/A |
| Mouse MC38 colon cancer cells | Cell bank of the Chinese academy of sciences | N/A |
| RAW264.7 cells | Cell bank of the Chinese academy of sciences | N/A |
| Mouse MB49 bladder cancer cells | Gifted from *Prof.* Longcheng Li, Peking Union Medical College Hospital | N/A |
| Mouse SJT-1601 lung carcinoma cell line | Gifted from *Dr.* Liufu Deng, Shanghai Jiao Tong University | N/A |
| **Primers and probes** | | |
| *Cyba* 5′-*GACCATTGCAAGTGAACACCC*-3′  5’-*AAATGAAGTGGACTCCACGCG*-3′ | | |
| *Cybb* 5′-*ATGGAGCGATGTGGACAGAAG*-3′  5′-*TAGATCACACTGGCAATGGCC-*3′ | | |
| *Ncf2* 5′-*TCTATCAGCTGGTTCCCACG*-3′  5′-*TGGCCTACTTCCAGAGAGGA*-3′ | | |
| *Cyba*-specific siRNA 5'-*GGACUCCCAUUGAGCCUAATT*-3' | | |
| *Cybb*-specific siRNA 5'- *GAUGGUAGCUUGGAUGAUATT*-3' | | |
| nonsense siRNA 5'-*UAUCAGCUUGUGGUUCAGUUCTT*-3' | | |
| **CUT&RUN** | | |
| *Cyba* 5′-CACTTCCTCAAACGGTGAGTG-3′  5’-AGAAACTAGAGACCTCAAGTAGGC-3′ | | |
| *Cybb* 5′-CACACCAGCTGCACAAACAG-3′  5′-TCCTGGCCTTCATTTGCCTC-3′ | | |
| *Ncf2* 5′-ATATGGCGAATGAAGGTGTCCC-3′  5′-TAGTGTATTTAAGGCCCAGCCC-3′ | | |
| **Deposited Data** | | |
| BMDM RNA sequencing | This Paper | GEO: GSEXXXX |
| NMIBC tumor RNA sequencing | This Paper | GEO: GSEXXXX |
| ATAC sequencing | This Paper | GEO: GSEXXXX |
| Mouse bladder tumor scRNA sequencing | Andrew W,et al.2025 | GEO: GSE295309 |
| Human Urine scRNA sequencing | Michelle A,et al.2024 | GEO: GSE267718 |
| **Software and algorithms** | | |
| FlowJo | Tree Star | RRID: SCR_008520 |
| Prism v.10.0 | GraphPad | RRID:SCR_005375 |
| ImageJ | NIH | RRID:SCR_003070 |
| Loupe Browser 7 | 10X GENOMICS | RRID:SCR_018555 |
| ImarisViewer 10.0.1 | Oxford Instruments |  |
| R statistical programming | N/A | RRID: SCR_001905 |
| Genomics Viewer (IGV) version 2.18.2 | N/A |  |
